# Predictors of discontinuation, efficacy, and safety of memantine treatment for Alzheimer’s disease: meta-analysis and meta-regression of 18 randomized clinical trials involving 5004 patients

**DOI:** 10.1186/s12877-018-0857-5

**Published:** 2018-07-24

**Authors:** Lídia Blanco-Silvente, Dolors Capellà, Josep Garre-Olmo, Joan Vilalta-Franch, Xavier Castells

**Affiliations:** 1 TransLab Research Group; Department of Medical Sciences, University of Girona, Girona, Spain; 2 Department of Medical Sciences, Faculty of Medicine, University of Girona, Emili Grahit, 77, 17003 Girona, Spain; 3 Girona Biomedical Research Institute (IdibGi), Parc Hospitalari Martí i Julià, Salt, Spain

**Keywords:** Alzheimer’s disease, Memantine, Discontinuation, Efficacy, Meta-analysis, Meta-regression

## Abstract

**Background:**

The risk-benefit relationship of memantine treatment for Alzheimer’s disease (AD) remains unclear. In addition, variability between the results of clinical trials has been observed. The aim of this study was to investigate the risk-benefit relationship of memantine treatment in patients with AD and to determine the predictor effect of patient, intervention, and study design related covariates.

**Methods:**

A systematic review and meta-analysis of double-blind, placebo controlled clinical trials was performed. Primary outcomes were all-cause discontinuation, discontinuation due to adverse events (AE) and efficacy on cognitive function. Odds ratio (OR) and standard mean difference (SMD) with 95% confidence intervals were calculated. Meta-regression was conducted to identify related covariates. Cochrane Collaboration tool was used to evaluate the risk of bias of included trials.

**Results:**

Eighteen studies involving 5004 patients were included. No differences between memantine and placebo were found for all-cause treatment discontinuation (OR=0.97 [0.82, 1.14]) and discontinuation due to AE (OR=1.18 [0.91, 1.53]). Memantine showed small improvement on cognitive function (SMD=0.15 [0.08, 0.22]). Baseline functional ability was positively associated with all-cause treatment discontinuation and discontinuation due to AE.

**Conclusions:**

Our study suggests that memantine has a very small efficacy on AD symptomatology and its safety profile is similar to that of placebo. No evidence of treatment discontinuation improvement with memantine is found, indicating a dubious risk-benefit relationship. No intervention characteristic or subgroup of patients clearly shows a significantly better risk-benefit relationship.

**PROSPERO registration:**

CRD42014015696.

**Electronic supplementary material:**

The online version of this article (10.1186/s12877-018-0857-5) contains supplementary material, which is available to authorized users.

## Background

Around 47 million people worldwide have dementia and, by 2030, it is expected to affect 75.6 million people, with Alzheimer’s disease (AD) being the most common cause [1]. AD is a neurodegenerative disorder characterised by cognitive impairment, behaviour disturbances and functional disability. AD incurs significant health and community care costs [2], and both cognitive and functional decline are associated with caregiver outcomes [3].

Memantine is a non-competitive N-methyl-D-aspartate (NMDA) receptor antagonist that has shown a neuroprotective effect in some studies [4, 5]. It is approved for the treatment of moderate to severe AD [6, 7], and some guidelines support using it in combination with a cholinesterase inhibitor (ChEI) [8] whereas others do not recommend it because important gaps in the evidence exist [9]. Since memantine is the only drug authorized for treating patients with moderate-severe AD, its frequent prescription is unsurprising [10–13]. Nevertheless, the risk-benefit relationship associated with it remains unclear for several reasons. Firstly, the efficacy of memantine has been studied fundamentally on the severity of AD symptoms. However, the use of this outcome is arguable because of its dubious clinical relevance. For this reason, pragmatic outcomes like institutionalisation or mortality have been recommended [9]. In addition, the validity of ‘symptom improvement’ may be hampered by blinding failure and attrition bias [14]. Secondly, memantine has been associated with several side effects such as dizziness, headaches, constipation, somnolence, hypertension and agitation, some of which may be serious [6, 7, 13]. In this context, it is difficult to weigh memantine’s efficacy against its safety. This problem can be partly overcome using “all-cause treatment discontinuation”, a pragmatic outcome that reflects therapeutic benefits in relation to undesirable effects [15]. In addition, it is unaffected by attrition bias as this outcome has no missing data. Treatment discontinuation has been used previously in the field of AD [15–17] and other disorders [18–20].

Another issue that further complicates the assessment of memantine’s risk-benefit relationship is the variability of results between different clinical trials. While some studies show positive findings on cognitive symptoms [21, 22] and discontinuation rate, others find no differences from placebo [23–27]. Between-study variability can be determined by means of meta-analysis, which allows for calculating the proportion of the variability in effect estimates that is due to heterogeneity rather than chance [28]. The presence of statistical heterogeneity reflects inconsistency, and this reduces the confidence in the meta-analysis findings and the strength of clinical recommendations derived from them. Determining the sources of between-study variability enables clustering the studies in groups with more consistent results for which specific clinical recommendations should be made [29]. Meta-regression can be used to determine the sources of between-study variability, which could be related to the following: (1) the study design, such as whether there is a lead-in period [30] and the number of study sites [31]; (2) the intervention studied, for example dose [32, 33] and length of treatment [34, 35]; (3) patient characteristics, for example age [36] and severity of the disease [37]; and (4) study sponsorship [19, 38]. Identifying these factors can help tailor treatment with memantine for patients with AD and guide future research.

Previous meta-analyses have analysed the efficacy and safety of memantine for AD [17, 39–48]; however, none has used all-cause discontinuation to assess the risk-benefit treatment relationship, nor has investigated extensively the sources of between-study variability in different outcomes of discontinuation, efficacy, and safety. To fill this gap, this study aims to (1) evaluate the risk-benefit relationship of memantine in patients with AD using all-cause treatment discontinuation as the primary outcome; (2) determine the predictor effect of study design, patient, and intervention related covariates on discontinuation, efficacy, and safety outcomes by performing meta-analysis and meta-regression.

## Methods

### Study design and search strategy

We conducted a systematic review and meta-analysis (SRMA) of double-blind, randomized, placebo-controlled, parallel-group clinical trials (RPCCT) that investigated the effect of memantine (dose 5 to 23 mg/day) in monotherapy, or in combination with ChEI in AD patients. The length of intervention was at least 12 weeks. We excluded articles in languages other than English, Spanish, Italian, French and Portuguese and studies published only as conference abstracts. The study protocol was registered at the International Prospective Register of Systematic Reviews (PROSPERO): CRD42014015696. The Preferred Reporting Items for Systematic Reviews and Meta-analysis (PRISMA) guidelines [49] were followed (see Additional file 1: Table S1).

The following databases were searched to identify studies meeting our inclusion criteria: Medline, Cochrane Central Register of Controlled Trials, PsycINFO, ISI Web of Knowledge, www.clinicaltrials.gov, www.clinicaltrialsregister.eu, www.controlled-trials.com. The search strategy is detailed in the (Additional file 1: Table S2). Reference lists of previous systematic reviews [17, 39–48], pharmaceutical industry databases and reports from drug regulatory agencies such as European Medicines Agency and Food and Drug Administration were reviewed to identify further studies. The limit of the search was 01 February 2017.

#### Data extraction and quality assessment

Data extraction was carried out independently by two reviewers (LB, XC) and disagreements were discussed with a third reviewer (DC). Study authors and pharmaceutical companies were emailed to obtain unpublished data. The risk of bias of the RPCCT included was assessed using the Cochrane Collaboration tool. This tool evaluates the risk of bias based on the description and suitability of the following domains: sequence generation, allocation concealment, blinding, incomplete data, selective outcome reporting, and other biases. A judgement relating to the risk of bias is given for each domain in terms of ‘low’, ‘high’, or ‘unclear’ risk.

#### Study outcomes

The primary outcomes were 1) all-cause treatment discontinuation, defined as the proportion of patients who did not complete the study for some reason; 2) discontinuation due to adverse events (AE), defined as the proportion of patients who dropped out due to side effects; and 3) efficacy on cognitive function, defined as the improvement in cognitive symptoms, giving preference to the Alzheimer’s disease Assessment Scale-Cognitive subscale (ADAS-cog) [50], followed by Mini-Mental State Examination (MMSE) [51], and then the Severe Impairment Battery (SIB) [52].

The secondary outcomes were 1) discontinuation due to lack of efficacy (LoE), defined as the proportion of patients who did not complete the study for inefficacy; 2) efficacy on global change from baseline, giving priority to the Clinician Interview-Based Impression on Change-Plus Caregiver Input (CIBIC-Plus) [53] over the Clinical Global Impression (CGI) [54]; 3) efficacy on neuropsychiatric symptoms, with preference given to the Neuropsychiatric Inventory (NPI) [55] over the Behavioural Pathology in Alzheimer’s Disease Rating Scale (BEHAVE-AD) [56]; 4) efficacy on functional ability, giving preference to the Alzheimer’s Disease Cooperative Study Activities of Daily Living Inventory 19- or 23-item Scale (ADCS-ADL) [57] over the Disability Assessment for Dementia (DAD) [58]; 5) mortality, as the proportion of patients who died; 6) AE, defined as the proportion of patients experiencing any side effect; 7) serious adverse events (SAE), defined as the proportion of patients experiencing one or more SAE and 8) drug-related adverse event (DRAE), defined as the proportion of patients experiencing one or more AE considered related with drug intervention by the investigator during the study. We preferred intention to treat analysis data (ITT) to per-protocol (PP). For efficacy outcomes, we preferred changes scores to endpoint scores, and these to response rates.

The following covariates were collected: number of study sites (single vs multi-site); lead-in period (yes vs no); placebo lead-in period (yes vs no); type of statistical analysis (ITT vs non-ITT); memantine intervention (monotherapy vs combination with ChEI); dose (20 vs 28 mg/day); regimen (qd vs bid); dosage (fixed vs flexible); length of the intervention (weeks); age (years); gender (% women); AD baseline severity (mild, mild-moderate, moderate, moderate-severe, severe); baseline cognitive function (% scale maxima); baseline neuropsychiatric symptom severity (% scale maxima); baseline functional ability (% scale maxima) and study funding (independent vs industry). Regarding the type of statistical analysis carried out, we considered ITT approach when the number of patients included in the efficacy analyses was at least 95% of the total number of randomized patients, the others being non-ITT. Regarding memantine intervention, we considered that memantine was administered in combination with ChEI when more than 50% of patients received donepezil, galantamine or rivastigmine.

Baseline cognitive function, neuropsychiatric symptoms and functional ability were assessed using various scales. In order to standardize the baseline scores of these covariates, we calculated the percentage of scale maxima, which re-expresses the score as if the scale ranged from 0 to 100.

#### Statistical analysis

Odds ratio (OR) and 95% confidence intervals (CI) were calculated for dichotomous outcomes, and standardized mean difference (SMD) for continuous ones. For efficacy outcomes we used change scores, endpoint scores, and response rates as their combination has been shown to be valid [59, 60]. OR were re-expressed as SMD to allow them to be combined with continuous outcomes [61] (For an example on how we combined different efficacy scales and scores see Additional file 2: Table S3). A SMD of 0.2 was considered small, of 0.5 moderate, and SMD above 0.8 was considered large [62]. For outcomes where the efficacy was assessed using the same rating scale, mean difference (MD) was calculated. In RPCCT that compared memantine in monotherapy and in combination with ChEI vs. placebo, we analysed the effect of each intervention separately. However, the number of patients in the placebo group was divided by two to avoid over-counting [60]. Heterogeneity was assessed using the uncertainty factor *I*^*2*^, which measures the percentage of variance across studies that is due to heterogeneity rather than chance [62]. We combined OR and SMD by means of a random effects model [63], which takes into account both within- and between-study heterogeneity. The potential sources of heterogeneity were analysed using meta-regression [64] irrespective of the percentage of *I*^*2*^, because of the low sensitivity of the test. To quantify the proportion of variance explained by the covariate, we calculated the R^2^ index, which represents the ratio of explained variance to total variance [65]. All analyses were conducted using Comprehensive Meta-Analysis software (version 3) [66]. The full study dataset is provided in the (Additional file 2: Tables S4-S13).

Two sensitivity analyses were performed by 1) repeating the analysis after the exclusion of RPCCT with a high risk of bias in at least one domain; and 2) including the results of one pooled analysis [67], which reports the results of two Japanese clinical trials whose primary results could not be found (*post-hoc* analysis). Publication bias was assessed with funnel plots [68] and Begg’s [69] and Egger’s test [70].

## Results

### Study design, intervention and patient characteristics

**Fig. 1:**
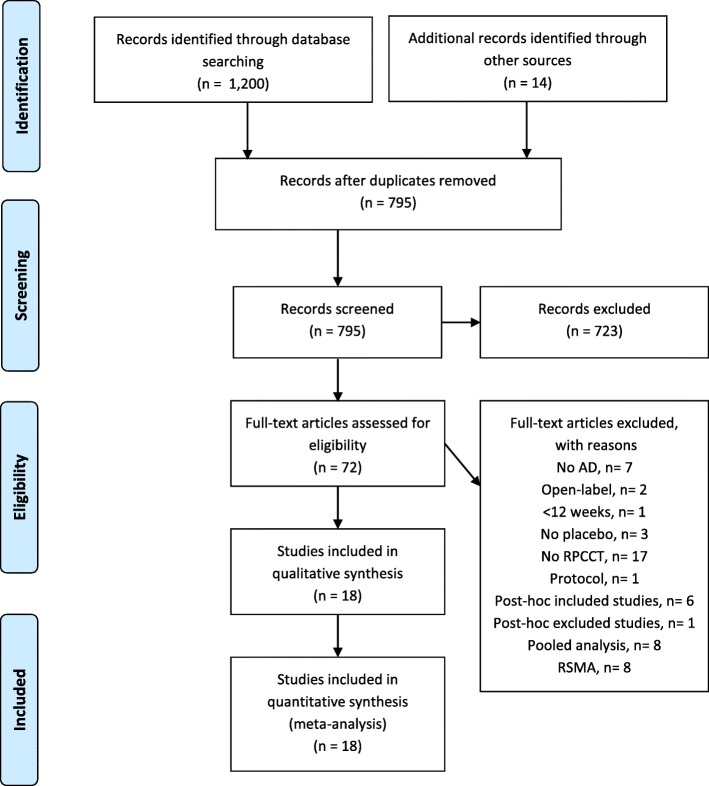
Preferred Reporting Items for Systematic Reviews and Meta-analyses (PRISMA) flow diagram

Eighteen studies were included (see Fig. 1 and Additional file 3: Table S14 and Table S15) involving nineteen memantine vs placebo comparisons. Table 1 shows study design, intervention and patients’ characteristics. Most studies were multi-site (77.8%), one third (33.3%) had a placebo lead-in period, and a high proportion of them (83.3%) had commercial sponsorship. Slightly over half (55.6%) of the studies included patients with moderate-severe AD. ITT was the most common statistical approach, except in neuropsychiatric symptoms, in which case non-ITT analysis prevailed (85.7%) (see Additional file 3: Table S16).

**Table 1:** Studies, interventions and patients’ characteristics, and risk of bias of included clinical trials

Studies	
Number of studies^a^	18
Number of drug-placebo comparisons	19
Number of patients/study (median) [range]	287 [13–677]
Multi-site studies (%)	77.8
Lead-in period (%)	33.3
Placebo lead-in period (%)	100
AD severity (%)	
Mild	0
Mild-moderate	33.3
Moderate	11.1
Moderate-severe	55.6
Severe	0
Study funding (%)	
Independent	16.7
Industry	83.3
ITT statistical analysis (%)	
Discontinuation outcomes	100
Efficacy cognitive function	56.2
Efficacy global change	70.0
Efficacy neuropsychiatric symptoms	14.3
Efficacy functional ability	60.0
Safety outcomes	100
Intervention^b^	
Monotherapy (%)	57.9
Combination ChEI (%)	42.1
Dose (%)	
20 mg	94.4
28 mg	5.6
Dosage (%)	
Fixed	94.4
Flexible	5.6
Regimen (%)	
qd	31.6
bid	68.4
Length (mean) [range]	39.1 [12–208]
12–24weeks (%)	15.8
v≥24–36 weeks (%)	52.6
≥36 weeks (%)	31.6
Patients	
Number of patients	5004
Age (mean) [range]	75.8 [65.2–84.6]
<75 years (%)	33.3
≥75–77 years (%)	38.9
≥77–79 years (%)	22.2
≥80 years (%)	5.6
Women (%) [range]	59.5 [2.9–86.7]
Baseline cognitive function (mean) ^c^[range]	45.6 [24.3–72.4]
Baseline severity neuropsychiatric symptoms (mean) ^c^[range]	11.3 [4.4–25.4]
Baseline functional ability (mean) ^c^[range]	65.7 [50.2–79.6]
Scales of efficacy assessment^a^	
Cognitive function	
ADAS-Cog (%)	35.3
MMSE (%)	41.2
SIB (%)	23.5
Global change	
CIBIC-Plus (%)	80.0
CGI (%)	20.0
Neuropsychiatric symptoms	
NPI (%)	100
Functional ability	
ADCS-ADL_19_ (%)	60.0
ADCS-ADL_23_ (%)	40.0
High risk of bias^d^	
Discontinuation outcomes	0
Efficacy cognitive function	17.6
Efficacy global change	10.0
Efficacy neuropsychiatric symptoms	20.0
Efficacy functional ability	10.0
Safety outcomes	0

*Abbreviations*: *AD* Alzheimer’s disease, *ADAS-Cog* Alzheimer’s disease Assessment Scale-Cognitive subscale, *ADCS-ADL* Alzheimer’s Disease Cooperative Study Activities of Daily Living Inventory 19- or 23-item scale, *bid* twice a day, *CGI* Clinical Global Impression, *CIBIC-Plus* Clinician Interview-Based Impression on Change-Plus Caregiver Input, *ITT* intention to treat, *MMSE* Mini-Mental State Examination, *NPI* Neuropsychiatric Inventory, *SIB* Severe Impairment Battery, *qd* once a day

^a^One RPCCT included with factorial design

^b^Proportion of drug-placebo comparisons

^c^As a percentage of scale maxima (0–100)

^d^Proportion of comparisons with high risk of bias for each outcome

Regarding interventions, memantine in monotherapy was investigated in 11 studies and in combination with ChEI in 8 studies. One trial [71] had a factorial design and studied memantine and donepezil both alone and combined. All studies but one [21] investigated 20mg/day of memantine in a fixed dosage. The majority of studies investigating memantine in monotherapy used a twice daily regimen (81.8%), whereas a once daily regimen was more frequent in studies investigating memantine in combination with ChEI (62.5%). The length of the intervention ranged from 12 to 208 weeks, with a mean of 39 weeks. It was longer in studies investigating memantine in combination with ChEI (52.5 weeks) than in monotherapy (30.5 weeks).

A total of 5004 patients were included in the study. The mean age was 75.8years and over half (59.5%) were women. Regarding AD severity, patients showed moderate cognitive and neuropsychiatric and functional impairment.

MMSE was the most frequently used scale to evaluate cognitive function (41.2%), followed by ADAS-Cog (35.2%) and SIB (23.5%). CIBIC-Plus was the most commonly used instrument to assess global change (80%). All studies analysed used NPI for neuropsychiatric symptoms, and ADCS-ADL for functional ability.

### Risk of bias

None of the studies scored “high risk of bias” neither for discontinuation nor for safety outcomes. For efficacy outcomes, two studies were deemed to have a high risk of attrition bias. One of them showed differences in discontinuation rate between study groups [22]. The other study [71] had a notable discontinuation rate and performed an analysis *per protocol* (see Additional file 4: Figs. S1, S2 and Table S17).

### Meta-analysis and effect of covariates

Table 2 shows the effect of memantine on study outcomes, and Table 3 the effect of study design-, intervention-, and patient-covariates.

**Table 2:** Effect of memantine on discontinuation, efficacy and safety outcomes in patients with Alzheimer’s disease

	N^a^	OR	95%CI	*I*^*2*^ (%)
All-cause discontinuation	18	0.97	0.82, 1.14	14.9
Discontinuation due to AE	14	1.18	0.91, 1.53	34.4
Discontinuation due to LoE	7	0.40	0.18, 0.87	0
	N	SMD	95%CI	*I*^*2*^ (%)
Cognitive function	17	0.15	0.08, 0.22	24.3
Global change	10	0.16	0.08, 0.24	29.3
Neuropsychiatric symptoms	15	0.16	0.09, 0.24	27.2
Functional ability	10	0.07	−0.02, 0.15	14.7
	N	OR	95%CI	*I*^*2*^ (%)
Proportion patients AE	6	1.05	0.88, 1.25	0
Proportion patients SAE	10	0.89	0.70, 1.13	18.3
Mortality	13	1.03	0.74, 1.44	0

*AE* adverse event, *ChEI* cholinesterase inhibitor, *CI* confidence interval, *I*^*2*^ heterogeneity, *LoE* lack of efficacy, *N* number of memantine-placebo comparisons, *OR* odds ratio, *SAE* severe adverse event

^a^One study included had a factorial design

**Table 3:** Meta-regression analyses of study design-, intervention-, and patient-related covariates associated with study outcomes

	All-cause discontinuation			Discontinuation due to AE			Cognitive function			Global change			Neuropsychiatric symptoms			Functional ability			Proportion patients SAE		
	N	Constant (95%CI) Log OR (95%CI)	R^2^	N	Constant (95%CI) Log OR (95%CI)	R^2^	N	Constant (95%CI) Diff SMD (95%CI)	R^2^	N	Constant (95%CI) Diff SMD (95%CI)	R^2^	N	Constant (95%CI) Diff SMD (95%CI)	R^2^	N	Constant (95%CI) Diff SMD (95%CI)	R^2^	N	Constant (95%CI) Log OR (95%CI)	R^2^
Study site																					
Single site (ref.)	18	−0.026 (−1.145, 1.093)	0	14	2.314 (− 0.927, 5.555)	0.11	17	0.157 (−0.441, 0.754)	0	–	–	–	15	0.093 (−0.758, 0.944)	0	–	–	–	–	–	–
Multi-site		−0.007 (−1.139, 1.125)			−2.162 (−5.414, 1.089)			−0.007 (− 0.610, 0.595)		–	–	–		0.072 (− 0.783, 0.926)		–	–	–	–	–	–
Lead-in period																					
No (ref.)	18	0.071 (− 0.147, 0.290)	0.27	14	0.292 (−0.073, 0.657)	0	17	0.134 (0.033, 0.236)	0	10	0.104 (−0.037, 0.245)	0.05	15	0.185 (0.082, 0.288)	0	10	0.057 (−0.075, 0.189)	0	10	−0.131 (− 0.524, 0.262)	0
Yes		−0.223 (− 0.543, 0.097)			−0.267 (− 0.799, 0.265)			0.033 (− 0.117, 0.182)			0.087 (− 0.084, 0.258)			−0.046 (− 0.198, 0.107)			0.016 (− 0.160, 0.191)			0.021 (− 0.505, 0.546)	
Placebo lead-in period																					
No (ref.)	18	0.071 (− 0.147, 0.290)	0.27	14	0.292 (−0.073, 0.657)	0	17	0.134 (0.033, 0.236)	0	10	0.104 (−0.037, 0.245)	0.05	15	0.185 (0.082, 0.288)	0	10	0.057 (−0.075, 0.189)	0	10	−0.131 (− 0.524, 0.262)	0
Yes		−0.223 (− 0.543, 0.097)			−0.267 (− 0.799, 0.265)			0.033 (− 0.117, 0.182)			0.087 (− 0.084, 0.258)			−0.046 (− 0.198, 0.107)			0.016 (− 0.160, 0.191)			0.021 (−0.505, 0.546)	
Statistical analysis																					
ITT (ref.)	–	–	–	–	–	–	17	0.162 (0.069, 0.256)	0	10	0.192 (0.100, 0.283)	0.15	15	0.006 (−0.153, 0.164)	0.72	10	0.102 (0.002, 0.202)	0.10	–	–	–
Non-ITT		–			–			−0.036 (− 0.190, 0.118)			−0.104 (− 0.282, 0.073)			0.089 (0.011, 0.358)*			− 0.104 (− 0.273, 0.066)			–	
Intervention																					
Monotherapy (ref.)	18	0.054 (−0.195, 0.302)	0	14	0.244 (−0.166, 0.654)	0	17	0.177 (0.068, 0.286)	0	10	0.168 (0.040, 0.295)	0	15	0.212 (0.103, 0.321)	0.02	10	0.089 (− 0.038, 0.216)	0	10	−0.032 (− 0.407, 0.343)	0
Combination ChEI		−0.157 (− 0.492, 0.178)			−0.138 (− 0.689, 0.413)			− 0.052 (− 0.202, 0.097)			−0.009 (− 0.181, 0.164)			− 0.090 (− 0.236, 0.057)			−0.043 (− 0.217, 0.131)			− 0.163 (− 0.677, 0.350)	
Dose																					
20 mg/day (ref.)	18	−0.049 (− 0.230, 0.132)	0	14	0.126 (−0.154, 0.406)	0	17	0.141 (0.061, 0.220)	0	10	0.148 (0.059, 0.237)	0	15	0.160 (0.079, 0.242)	0	10	0.064 (−0.032, 0.159)	0	10	−0.182 (− 0.428, 0.063)	0.50
28 mg/day		0.136 (−0.388, 0.661)			0.376 (−0.478, 1.229)			0.072 (− 0.162, 0.306)			0.102 (− 0.126, 0.330)			0.038 (−0.205, 0.280)			0.019 (−0.240, 0.277)			0.470 (−0.207, 1.147)	
Dosage																					
Fixed (ref.)	18	−0.049 (− 0.230, 0.132)	0	14	0.126 (− 0.154, 0.406)	0	17	0.141 (0.061, 0.220)	0	10	0.148 (0.059, 0.237)	0	15	0.160 (0.079, 0.242)	0	10	0.064 (−0.032, 0.159)	0	10	−0.182 (− 0.428, 0.063)	0.50
Flexible		0.136 (−0.388, 0.661)			0.376 (−0.478, 1.229)			0.072 (−0.162, 0.306)			0.102 (−0.126, 0.330)			0.038 (−0.205, 0.280)			0.019 (−0.240, 0.277)			0.470 (−0.207, 1.147)	
Regimen																					
QD (ref.)	18	0.057 (−0.206, 0.320)	0	14	0.263 (−0.209, 0.735)	0	17	0.077 (−0.025, 0.180)	0.48	10	0.109 (−0.030, 0.248)	0	15	0.123 (0.010, 0.237)	0	10	−0.023 (− 0.152, 0.106)	0.36	10	0.050 (−0.391, 0.491)	0
BID		−0.149 (− 0.487, 0.189)			−0.143 (− 0.718, 0.432)			0.125 (− 0.011, 0.260)			0.084 (−0.089, 0.257)			0.070 (−0.080, 0.219)			0.137 (−0.024, 0.297)			−0.245 (− 0.776, 0.286)	
Length (weeks)																					
Intercept	18	−0.076 (− 0.296, 0.144)	0	14	0.124 (−0.225, 0.473)	0	17	0.145 (0.046, 0.243)	0	10	0.270 (−0.170, 0.709)	0	15	0.196 (0.101, 0.292)	0.01	10	0.045 (−0.062, 0.152)	0	10	−0.038 (− 0.361, 0.286)	0
		0.001 (−0.002, 0.004)			0.001 (−0.004, 0.006)			0.000 (−0.002, 0.002)			−0.005 (− 0.024, 0.014)			−0.001 (− 0.002, 0.001)			0.001 (− 0.001, 0.002)			0.001 (−0.005, 0.002)	
Age (years)																					
Intercept	18	−1.674 (−6.563, 3.216)	0	14	−0.115 (−7.371, 7.141)	0	17	−0.084 (−2.235, 2.068)	0	10	−0.586 (−3.310, 2.137)	0	15	−1.287 (−3.409, 0.836)	0.12	10	−1.304 (−4.816, 2.208)	0	10	4.223 (−6.002, 14.448)	0
		0.021 (−0.042, 0.085)			0.004 (−0.091, 0.098)			0.003 (−0.025, 0.031)			0.010 (−0.026, 0.045)			0.019 (−0.009, 0.047)			0.018 (−0.028, 0.064)			−0.057 (− 0.190, 0.077)	
Women (%)																					
Intercept	18	0.117 (−0.428, 0.662)	0	14	0.359 (−0.463, 1.182)	0	17	0.128 (−0.147, 0.403)	0	10	−0.099 (− 0.880, 0.681)	0	15	−0.038 (− 0.293, 0.216)	0.35	10	0.076 (− 0.215, 0.367)	0	10	−0.378 (− 0.950, 0.194)	0
		−0.003 (− 0.012, 0.006)			−0.003 (− 0.017, 0.010)			0.0004 (− 0.004, 0.005)			0.004 (−0.008, 0.016)			0.003 (−0.001, 0.008)			−0.0002 (− 0.005, 0.005)			0.005 (−0.005, 0.015)	
AD baseline severity																					
Mild-moderate (ref.)	18	0.117 (−0.170, 0.404)	0.19	14	0.338 (−0.063, 0.839)	0.03	17	0.125 (−0.004, 0.254)	0	10	0.139 (−0.007, 0.286)	0	15	0.089 (−0.032, 0.210)	0.24	10	0.029 (−0.03, 0.160)	0	10	−0.051 (− 0.414, 0.312)	0
Moderate-severe		−0.219 (− 0.566, 0.127)			−0.334 (− 0.887, 0.219)			0.036 (− 0.122, 0.194)			0.036 (−0.144, 0.215)			0.110 (−0.039, 0.259)			0.063 (−0.108, 0.235)			−0.142 (− 0.663, 0.380)	
Baseline cognitive function (mean)																					
Intercept	18	−0.298 (− 0.797, 0.202)	0.12	14	−0.299 (−1.080, 0.481)	0.09	17	0.261 (0.032, 0.490)	0.02	10	0.215 (−0.068, 0.499)	0	15	0.387 (0.185, 0.589)	0.88	10	0.198 (−0.071, 0.467)	0.01	10	−0.125 (−1.011, 0.761)	0
		0.006 (−0.005, 0.016)			0.010 (−0.006, 0.026)			−0.003 (− 0.007, 0.002)			−0.001 (− 0.007, 0.005)			− 0.005 (− 0.009, − 0.001)**			−0.003 (− 0.008, 0.003)			0.0001 (−0.016, 0.017)	
Baseline neuropsychiatric symptoms severity (mean)																					
Intercept	15	−0.101 (− 0.530, 0.339)	0	12	0.163 (−0.505, 0.831)	0	15	0.210 (0.023, 0.397)	0	9	0.228 (−0.009, 0.466)	0	15	0.010 (−0.174, 0.193)	0.37	10	0.204 (−0.038, 0.447)	0.09	9	−0.178 (− 0.910, 0.555)	0
		0.006 (−0.027, 0.040)			0.0003 (−0.051, 0.052)			−0.005 (− 0.020, 0.010)			−0.006 (− 0.024, 0.012)			0.013 (− 0.002, 0.028)			−0.013 (− 0.034, 0.008)			0.010 (− 0.063, 0.084)	
Baseline functional ability (mean)																					
Intercept	11	−1.897 (−3.685, −0.110)	0.68	11	−2.536 (−5.183, 0.111)	0.47	12	0.140 (−0.492, 0.771)	0	8	0.040 (−0.413, 0.492)	0	10	0.461 (−0.253, 1.175)	0	10	0.702 (−0.061, 1.466)	0.44	8	−0.831 (−2.931, 1.269)	0
		0.028 (0.001, 0.055)*			0.041 (0.001, 0.081)*			0.000 (−0.010, 0.010)			0.002 (−0.006, 0.009)			−0.005 (− 0.016, 0.006)			−0.010 (− 0.021, 0.002)			0.011 (− 0.020, 0.042)	
Type of scale																					
Intercept (ref.)	–	–	–	–	–	–	17	ADAS-Cog 0.138 (0.003, 0.272)	0	10	CIBIC-Plus 0.151 (0.062, 0.241)	0	–	–	–	–	–	–	–	–	–
	–	–	–	–	–	–		MMSE 0.019 (−0.176, 0.214)			CGI 0.087 (−0.154, 0.328)		–	–	–	–	–	–	–	–	–
								SIB 0.016 (−0.173, 0.204)													
Sponsor																					
Independent (ref.)	18	0.133 (−0.275, 0.541)	0	14	0.306 (−0.524, 1.136)	0	17	0.183 (−0.019, 0.385)	0	–	–	–	15	0.178 (−0.026, 0.381)	0	10	0.151 (−0.136, 0.439)	0	10	−0.343 (− 0.921, 0.235)	0
Industry		−0.199 (− 0.645, 0.248)			−0.155 (−1.035, 0.725)			−0.040 (− 0.257, 0.178)		–	–	–		− 0.016 (− 0.236, 0.204)			−0.093 (− 0.394, 0.208)			0.274 (− 0.366, 0.914)	

^a^Type of scale used to evaluate the efficacy. Cognitive function: ADAS-Cog, MMSE or SIB; global change: CIBIC-Plus or CGI; neuropsychiatric symptoms: NPI; functional ability: ADCS-ADL

*Statistically significant effect (*p*-value ≤0.05) ** statistically significant effect (p-value ≤0.01)

*AD* Alzheimer’s disease, *AE* adverse event, *CI* confidence interval, *Diff SMD* difference of standardized mean difference, *ITT* intention to treat analysis, *LoE* lack of efficacy, *Log OR* Log odd ratio, *N* number of memantine-placebo comparisons, *NA* not applicable, *PP* per-protocol analysis, *R*^*2*^ proportion of variance explained by the covariate, *SAE* serious adverse event

#### Discontinuation outcomes

**Fig. 2:**
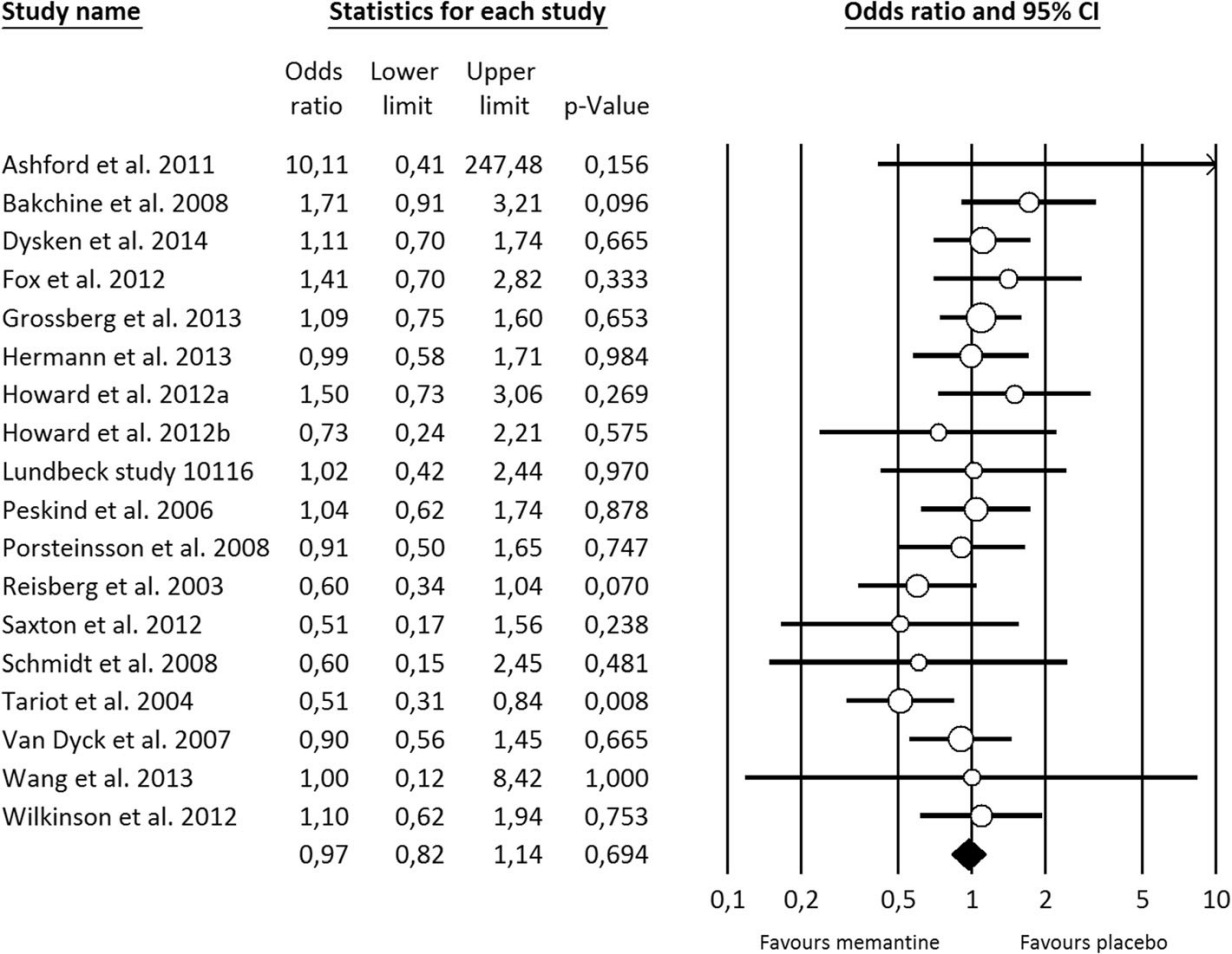
Forest plot of meta-analysis pooled effect memantine treatment on all-cause discontinuation

**Fig. 3:**
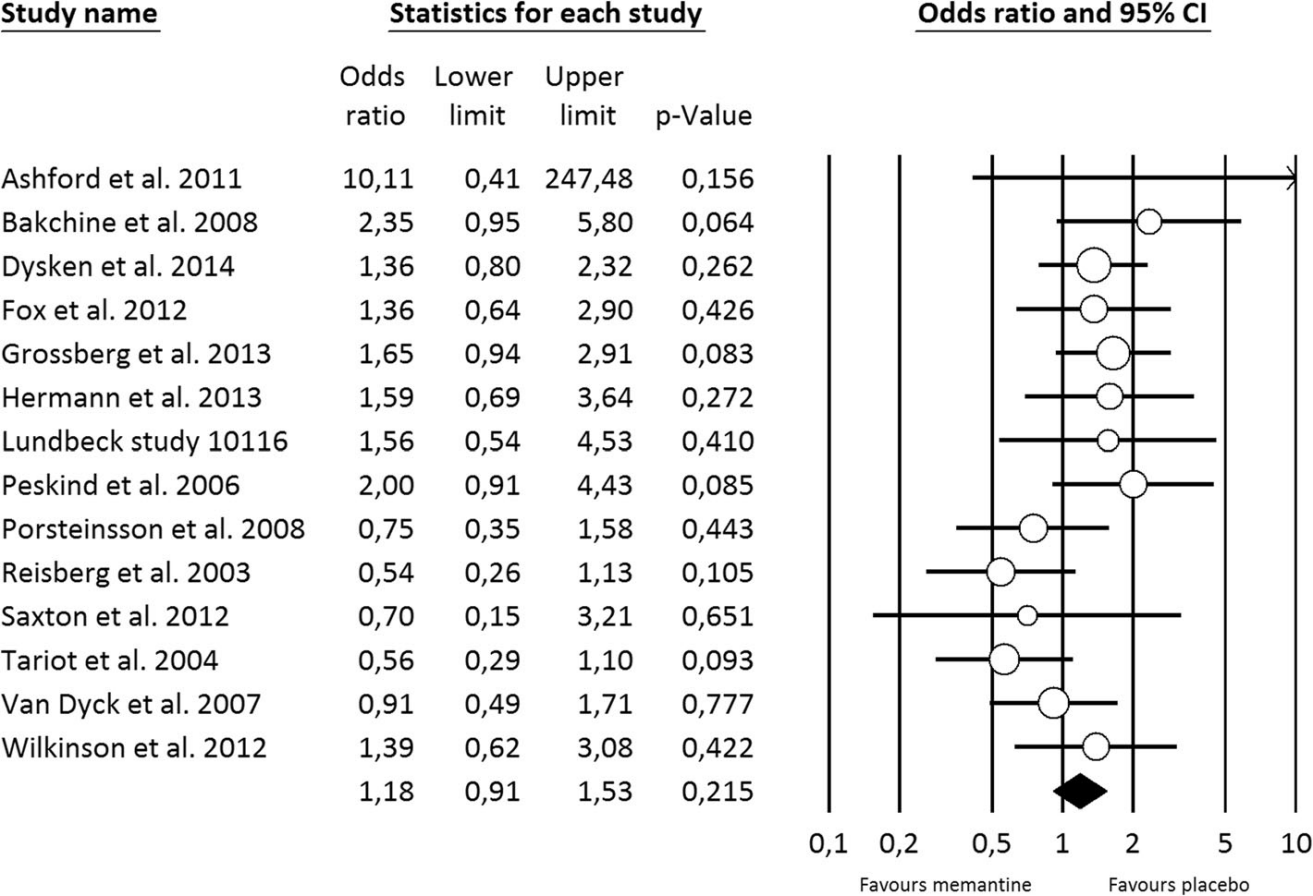
Forest plot of meta-analysis pooled effect memantine treatment on discontinuation due to AE

**Fig. 4:**
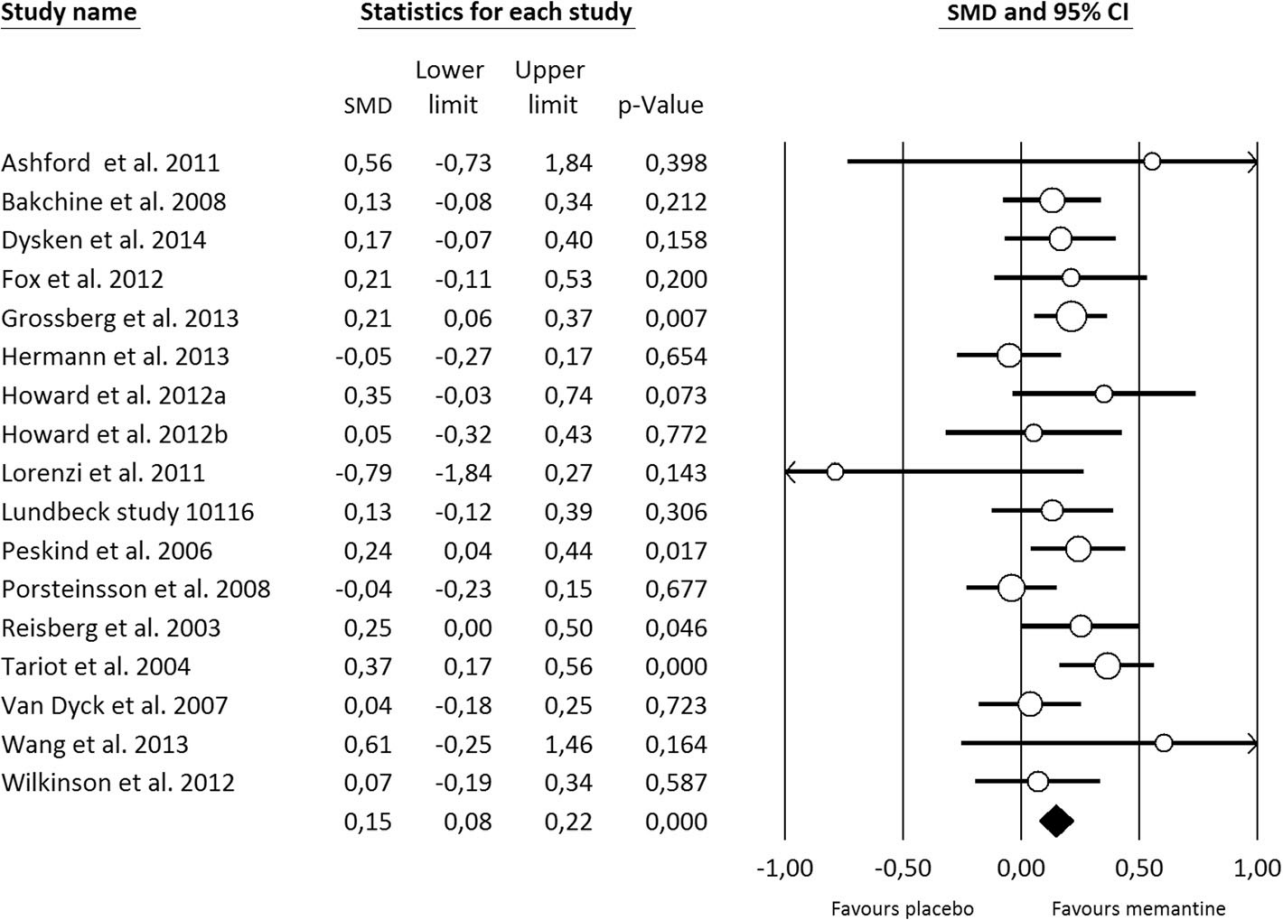
Forest plot of meta-analysis pooled effect of memantine treatment on cognitive function

Regarding all-cause treatment discontinuation, 4989 patients from seventeen studies were included in the analysis. The discontinuation rate was relatively low amongst patients receiving either memantine or placebo (18.2% vs 19.4%), and no statistically significant differences were found (OR=0.97 [0.82, 1.14], Fig. 2). The statistical heterogeneity was low (*I*^*2*^=14.9%) and the meta-regression analysis showed that baseline functional ability was positively associated with all-cause discontinuation (Log OR=0.028 [0.001, 0.055]), explaining 68% of the variability observed (Fig. 3). For discontinuation due to AE, no statistically significant differences were observed from placebo (14 memantine vs placebo comparisons; 4632 patients; OR=1.18 [0.91, 1.53], Fig. 4). A moderate statistical heterogeneity was found (*I*^*2*^=34.4%) and the baseline functional ability was also positively correlated with this outcome (Log OR=0.041 [0.001, 0.081]), explaining 47% of the variability (Fig. 3). Memantine showed a better outcome than placebo on discontinuation due to LoE (7 memantine vs placebo comparisons; 3015 patients; OR=0.40 [0.18, 0.87], Additional file 5: Fig. S3). Neither statistical heterogeneity nor statistically significant effect of any covariates were found (see Additional file 5: Table S18). Nevertheless, few studies provided data on this outcome.

#### Efficacy outcomes

**Fig. 5:**
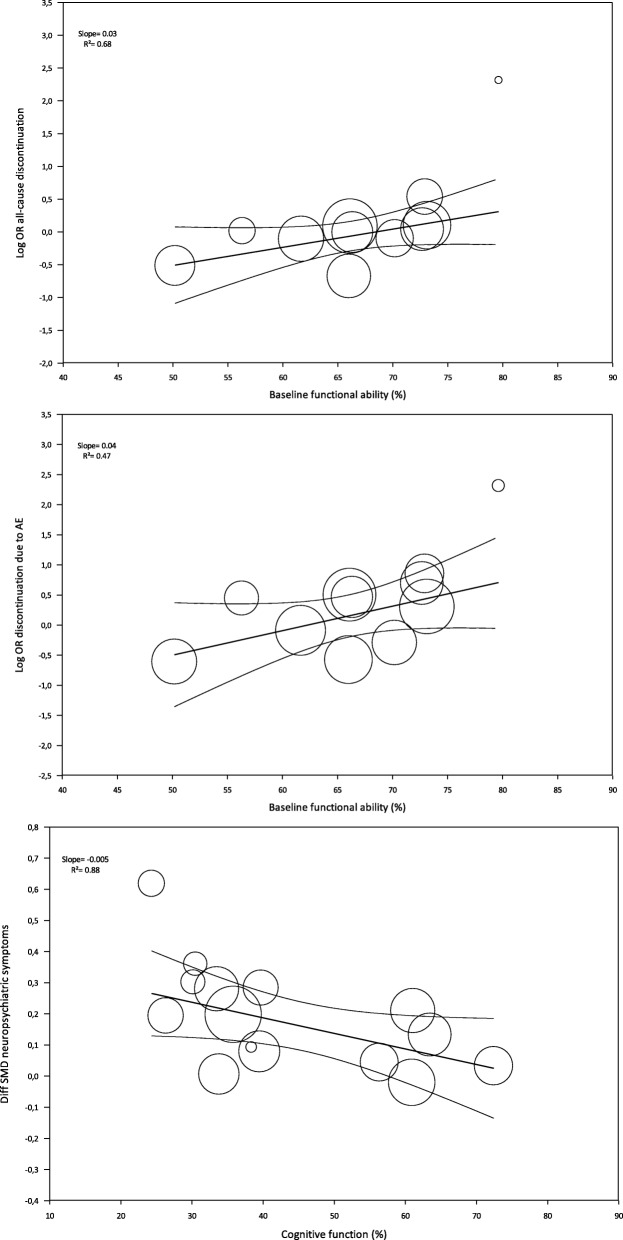
Scatterplots of covariates related to study outcomes. The effect of baseline functional ability on all-cause discontinuation (Top), the effect of baseline functional ability on discontinuation due to AE (Middle) and the effect of baseline cognitive function on neuropsychiatric symptoms (Bottom)

Regarding efficacy, when compared to placebo, memantine showed a small improvement on cognitive function (16 memantine vs placebo comparisons) studies; 4336 patients; SMD=0.15 [0.08, 0.22], Fig. 5), global symptomatology (10 memantine vs placebo comparisons; 4169 patients; SMD=0.16 [0.08, 0.24], Additional file 5: Fig. S4), and neuropsychiatric symptoms (14 memantine vs placebo comparisons; 5011 patients; SMD=0.16 [0.09, 0.24], Additional file 5: Fig. S5). Since all the studies in the analysis used the NPI scale, we calculated an MD of 2.2 points. Conversely, no differences were found regarding functional ability (10 memantine vs placebo comparisons; 4067 patients; SMD=0.07 [− 0.02, 0.15], Additional file 5: Fig. S6). For all efficacy outcomes, heterogeneity was low (cognitive function *I*^2^=24.3%; global change *I*^2^=29.3%; neuropsychiatric symptoms *I*^2^=27.2%; and functional ability *I*^2^=14.7%). Meta-regression analysis found that two covariates were correlated with neuropsychiatric symptom severity: baseline cognitive function and type of statistical analysis. Baseline cognitive function was negatively associated with neuropsychiatric symptoms (Diff SMD=− 0.005 [− 0.009, − 0.001]) accounting for 88% of variability (Fig. 4). The studies that used a non-ITT analysis showed a larger effect size than those using an ITT approach (Diff SMD=0.089 [0.011, 0.358]), representing 72% of the variance observed. No covariate was found to modify the effect of memantine on the remaining efficacy outcomes (Additional file 5: Table S18).

It is important to highlight that similar efficacy was found for memantine in both monotherapy and in combination with ChEI. No statistically significant differences were observed between the effect of memantine in monotherapy, or in combination with ChEI, on any efficacy outcome analysed (see Additional file 5: Table S19).

#### Safety outcomes

In relation to safety, no statistically significant differences were found in AE (6 memantine vs placebo comparisons; 2425 patients; OR=1.05 [0.88, 1.25], Additional file 5: Fig. S7) nor in SAE outcome (10 memantine vs placebo comparisons; 3693 patients; OR=0.89 [0.70, 1.13], Additional file 5: Fig. S8), nor in mortality (12 memantine vs placebo comparisons; 4232 patients; OR=1.03 [0.74, 1.44], Additional file 5: Fig. S9). The results for DRAE were not analysed as only one study [72] provided data. Low heterogeneity was found for SAE (*I*^2^=18.3%). However, no heterogeneity was observed for discontinuation due to LoE, or AE and mortality. No covariate had a statistically significant effect on any safety outcome (Additional file 5: Table S18).

### Sensitivity analysis and publication bias

Two sensitivity analyses were performed. The first excluded studies with a high risk of bias [22, 71], and the second included the results of two clinical trials identified in a pooled analysis [67] whose primary report could not be found. Both analyses found similar results to the primary analysis (see Additional file 6: Table S20 and Table S21).

No evidence of publication bias was found as none of the study outcomes showed funnel plot asymmetry. These results were confirmed with Begg’s and Egger’s tests (Additional file 6: Fig. S10).

## Discussion

A modest number of RPCCT have investigated the efficacy of memantine for AD as monotherapy or in combination with ChEI. Most studies had an unclear methodological quality fundamentally because it is doubtful whether blinding can be guaranteed, and because the possibility of attrition bias cannot be ruled out. We have found no difference between memantine and placebo on treatment discontinuation. It must be acknowledged that this is not a favourable outcome because, ideally, any symptomatic treatment should demonstrate a lower treatment discontinuation rate than placebo, as this would indicate that the improvement of symptoms outweighs side effects. Regarding discontinuation due to AE, no differences from placebo were observed. The statistical heterogeneity of these two outcomes of discontinuation is small-moderate, and a large proportion of between-study variance is explained by the patients’ baseline functional ability, which is associated with better outcome on all-cause discontinuation and discontinuation due to AE. A possible explanation is that, since patients with lower baseline functional ability are more impaired, they may be less sensitive to AE, or to report them less frequently, leading to lower discontinuation due to AE and a lower rate of all-cause treatment discontinuation.

Memantine is slightly more efficacious than placebo on cognitive function, global symptomatology, neuropsychiatric symptoms and discontinuation due to inefficacy. This finding is consistent with previous SRMA [17, 40–42, 48]. In addition, in accordance with Farrimond et al. [40], memantine does not improve functional ability.

Overall, these findings cast doubt on the clinical relevance of memantine’s efficacy for AD. Our study differs from others that reach more optimistic conclusions [17, 42, 45, 48], probably because their interpretation overlooks the effect size of the intervention, which is low to very small in all efficacy domains [73].

Between-study variability of efficacy outcomes is small. Two covariates modify the effect of memantine effect on neuropsychiatric symptoms. Firstly, one related with patient characteristics; the baseline cognitive function in the following way: patients with greater cognitive function show less improvement in neuropsychiatric symptomatology. Nevertheless, it must be taken into account that this finding is of dubious clinical relevance because the effect size of memantine on neuropsychiatric outcomes is very small, and differences on baseline cognitive function might not change significantly this effect size. The second covariate that was found to modify the effect of memantine on neuropsychiatric symptoms was the type of statistical analysis; with studies using a non-ITT approach showing a larger effect size than those using an ITT approach. The overestimation of the effect size in trials deviating from an ITT approach is consistent with previous research [74]. This could be due to multiple factors such as differential exclusion of patients with severe disease or those who are not doing well in a particular study arm [75]. It is important to highlight that no intervention-related covariates modified the effect of memantine on any study outcome, indicating that the effect of memantine does not change across time, with higher doses or when it is administered in combination with ChEI.

The results of our covariate analysis contrast with those of Taro et al. [17]. In this study, the authors found that the MMSE scores at baseline and the proportion of male were associated with the efficacy on cognitive function, and the sample size and the study duration were correlated with the improvement on behavioural disturbances. In addition, they did not find an association between ITT approach and efficacy on neuropsychiatric symptoms. The differences could be explained by different reasons. Firstly, the authors performed meta-regression analyses for monotherapy and combination therapy studies separately, without taking into account if there were differences between these two subgroups. Secondly, in the study by Taro et al. [17], the baseline cognitive function was measured only with MMSE, while we also used the ADAS-cog scale. Thirdly, Taro et al. [17] included both open-label and double-blind clinical trials. The definition of the covariate “Type of statistical analysis”, differed between the study by Taro et al. [17] and our study. We considered that the analysis was non-ITT when the number of patients included in the analysis was less than 95% of randomized patients, otherwise it was ITT. In contrast, Taro et al. [17] compared ITT or full analysis set population versus observed case analysis.

Regarding safety, as in previous studies [17, 41–43, 46, 48], our results support that memantine has a similar safety profile as placebo, since no differences were observed on AE, SAE and mortality. It could be argued that patients with AD may underreport AE, leading to an overestimation of memantine safety. In addition, differences in the incidence of SAE could be not detected. This was probably due to the low number of patients included and the relatively short length in clinical trials.

Overall, these results suggest that memantine has a questionable risk-benefit relationship providing a weak support for using memantine to treat patients with AD. This contrasts with the widespread use of memantine [9–12], which can be explained by the lack of pharmacological alternatives for patients with severe AD; a condition that is a significant burden on patients’ caregivers [3, 76] and a considerable cost to society [77, 78].

### Limitations and strengths

This study has limitations with regards to internal validity. The biases of RPCCT included might also bias the results of our meta-analysis. In any case, it does not appear to influence our study, as excluding the trials with a high risk of bias yields similar results to the main analyses. Publication bias can also affect results, but we found no evidence that it has affected our study. The possibility of ecological bias [79] must also be born in mind when interpreting meta-regression analyses. In addition, as patients were not randomized to the categories investigated using meta-regression, the possibility of confounding cannot be ruled out. As multiple comparisons have been made, it is possible that the differences observed have been found by chance.

In addition, there are limitations relating to external validity. The length of the studies is shorter than the current treatment with memantine in a clinical setting [80]. Furthermore, the strict inclusion criteria hinder the extrapolation of our results to clinical practice, as patients with serious comorbid diseases, which are common in clinical practice, are excluded from participating in clinical trials [81]. This is particularly relevant in relation to drug safety, thus our study may underestimate the risks associated with the administration of memantine. Finally, a further limitation affecting study precision must be taken into account when interpreting the findings of the meta-regression. This is particularly relevant to the outcomes “discontinuation due to LoE” and “proportion of patients with AE”, given that only a low number of studies were included in the analysis for these outcomes.

Regarding the strengths of the study, this is a comprehensive investigation of the risk-benefit of memantine for AD as we have analysed several efficacy, safety and discontinuation outcomes, in addition to mortality. This is, to the best of our knowledge, the largest SRMA conducted to investigate extensively the sources of between-study heterogeneity. Our findings expand and complement the results of previous studies [17, 39–48], providing evidence of the unconvincing effect of memantine treatment in patients with AD. Furthermore, the registry in PROSPERO, the accurate quality assessment of included trials and the transparency of the data give value to our study [82].

## Conclusions

This study concludes that memantine has a very small efficacy on cognitive, global and neuropsychiatric symptoms but does not improve functional ability. Despite it has a similar safety profile to that of placebo, no evidence of treatment discontinuation improvement is found, indicating overall that the risk-benefit relationship for the treatment of patients with AD is dubious. Between study-variability is low to moderate, and no intervention characteristic or subgroup of patients clearly shows a significantly better risk-benefit relationship.

## Additional files

Additional file 1: PRISMA checklist and search strategies. We provide the PRISMA checklist (**Table S1**) and search strategies (**Table S2**). (DOCX 42 kb)

Additional file 2: Study dataset. We provide the data used (**Tables** **S3**-**S13**). (DOCX 34 kb)

Additional file 3: Characteristics of included clinical trials. We provide the references of included studies (**Table S14**) and their study-, intervention-, and patient-related characteristics (**Table S15** and **Table S16**). (DOCX 32 kb)

Additional file 4: Risk of bias of included clinical trials. We provide the risk of bias of included studies on different domains using the Cochrane Collaboration tool (**Figure S2** and **Figure. S3**) and the high risk of bias by study outcomes (**Table S17**). (DOCX 73 kb)

Additional file 5: Forest plot of secondary outcomes and additional results. We provide the forest plot of secondary outcomes (**Figure S3**, **Figure S4**, **Figure S5**, **Figure S6**, **Figure S7**, **Figure S8** and **Figure S9**), the results of meta-regression of some secondary outcomes not provided in the manuscript (**Table S18**) and the results of the meta-analysis of study outcomes by type of intervention (monotherapy vs. combination with ChEI) (**Table S19**). (DOCX 249 kb)

Additional file 6: Sensitivity analyses and publication bias. We provide the results of the two sensitivity analyses: by excluding studies with high risk of bias (**Table S20**) and by including the results of Nakamura et al. 2014 pooled analysis (**Table S21**); and the funnel plots of study outcomes (**Figure S10**). (DOCX 291 kb)

## Abbreviations

AD: Alzheimer’s disease
ADAS-cog: Alzheimer’s disease Assessment Scale-Cognitive subscale
ADCS-ADL: Alzheimer’s Disease Cooperative Study Activities of Daily Living Inventory 19- or 23-item Scale
AE: Adverse event
BEHAVE-AD: Behavioural Pathology in Alzheimer’s Disease Rating Scale
BID: Twice daily regimen
CGI: Clinical Global Impression
ChEI: Cholinesterase inhibitor
CI: Confidence interval
CIBIC-Plus: Clinician Interview-Based Impression on Change-Plus Caregiver Input
DAD: Disability Assessment for Dementia
DRAE: Drug-related adverse event
ITT: Intention to treat analysis data
LoE: Lack of efficacy
MMSE: Mini-mental State Examination
NICE: National Institute of Health and Care Excellence
NMDA: N-methyl-D-aspartate
NPI: Neuropsychiatric Inventory
OR: Odds ratio
PP: Per-protocol analysis
PRISMA: Preferred Reporting Items for Systematic Reviews and Meta-Analysis
PROSPERO: International Prospective Register of Systematic Reviews
QD: Once daily regimen
RPCCT: Randomized placebo-controlled clinical trial
SAE: Severe adverse event
SIB: Severe Impairment Battery
SMD: Standard mean difference
SRMA: Systematic review and meta-analysis
