# Supplementary material for: Predictors of discontinuation, efficacy, and safety of memantine treatment for Alzheimer’s disease: meta-analysis and meta-regression of 18 randomized clinical trials involving 5004 patients
Source: BMC Geriatr. 2018 Jul 24;18:168. doi: 10.1186/s12877-018-0857-5 (PMC6057050; doi:10.1186/s12877-018-0857-5)
Supplement: Supplementary file 2 — Study dataset. We provide the data used (Tables S3-S13). (DOCX 34 kb) [file 12877_2018_857_MOESM2_ESM.docx]

**Table S3** Example of combination of continuous and binary data of efficacy on global change

|  |  | Mean change from baseline | | | | | | Responders | | | | Effect size | | |
| --- | --- | --- | --- | --- | --- | --- | --- | --- | --- | --- | --- | --- | --- | --- |
|  | Scale | Memantine | SD | n | Placebo | SD | n | Memantine | n | Placebo | n | OR (95%CI) | SMD | SEM |
| Bakchine et al. 2007 | CIBIC-Plus | -4.12 | 1.31 | 268 | -4.19 | 1.16 | 135 | 84 | 318 | 34 | 152 | - | 0.06 | 0.11 |
| Saxton et al. 2012 | CGI | - | - | - | - | - | - | 52 | 133 | 35 | 124 | 1.63  (0.97, 2.76) | 0.27 | 0.15 |

SMD was calculated for efficacy on global change using Cohen’s d. In Bakchine *et al.* 2007, the scale used was Clinician Interview-Based Impression on Change-Plus Caregiver Input (CIBIC-Plus) and mean change scores was preferred on responders. In Saxton *et al.* 2012, the scale used was Clinical Global Impression (GCI) and no continuous data was available. Odds ratio (OR) was reexpressed as Standardized Mean Difference (SMD) to allow the combination with continuous data (Higgins et al. 2011b).

CI, Confidence interval; SEM, Standard Error of the Mean; n, number of patients included in the analysis.

**Table S4** Raw data used for all-cause discontinuation outcome

| Trial | Memantine | | Placebo | |
| --- | --- | --- | --- | --- |
|  | Event | Total | Event | Total |
| Ashford et al. 2011 | 3 | 7 | 0 | 6 |
| Bakchine et al. 2008 | 47 | 318 | 14 | 152 |
| Dysken et al. 2014 | 67 | 155 | 62 | 152 |
| Fox et al. 2012 | 25 | 74 | 21 | 79 |
| Grossberg et al. 2013 | 69 | 342 | 63 | 335 |
| Herrmann et al. 2013 | 31 | 182 | 32 | 187 |
| Howard et al. 2012a | 25 | 76 | 18 | 73 |
| Howard et al. 2012b | 6 | 73 | 8 | 73 |
| Kitamura et al. 2011 | 30 | 207 | 21 | 108 |
| Lorenzi et al. 2011 | 0 | 7 | 0 | 8 |
| Lundbeck study 10116 | 11 | 128 | 11 | 130 |
| Nakamura et al. 2011 | 29 | 218 | 33 | 208 |
| Peskind et al. 2006 | 36 | 201 | 35 | 202 |
| Porsteinsson et al. 2008 | 23 | 217 | 25 | 216 |
| Reisberg et al. 2003 | 29 | 126 | 42 | 126 |
| Saxton et al. 2012 | 5 | 136 | 9 | 129 |
| Schmidt et al. 2008 | 5 | 18 | 7 | 18 |
| Tariot et al. 2004 | 30 | 203 | 51 | 201 |
| Van Dyck et al. 2007 | 44 | 178 | 46 | 172 |
| Wang et al. 2013 | 2 | 13 | 2 | 13 |
| Wilkinson et al. 2012 | 30 | 134 | 30 | 144 |
| Overall | 547 | 3,013 | 530 | 2,732 |

**Table S5** Raw data used for discontinuation due to AE outcome

| Trial | Memantine | | Placebo | |
| --- | --- | --- | --- | --- |
|  | Event | Total | Event | Total |
| Ashford et al. 2011 | 3 | 7 | 0 | 6 |
| Bakchine et al. 2008 | 28 | 318 | 6 | 152 |
| Dysken et al. 2014 | 40 | 155 | 31 | 152 |
| Fox et al. 2012 | 19 | 74 | 16 | 79 |
| Grossberg et al. 2013 | 34 | 342 | 21 | 335 |
| Herrmann et al. 2013 | 15 | 182 | 10 | 187 |
| Lorenzi et al. 2011 | 0 | 7 | 0 | 8 |
| Lundbeck study 10116 | 9 | 128 | 6 | 130 |
| Nakamura et al. 2011 | 14 | 218 | 13 | 208 |
| Peskind et al. 2006 | 19 | 201 | 10 | 202 |
| Porsteinsson et al. 2008 | 13 | 217 | 17 | 216 |
| Reisberg et al. 2003 | 13 | 126 | 22 | 126 |
| Saxton et al. 2012 | 3 | 136 | 4 | 129 |
| Tariot et al. 2004 | 15 | 203 | 25 | 201 |
| Van Dyck et al. 2007 | 22 | 178 | 23 | 172 |
| Wilkinson et al. 2012 | 15 | 134 | 12 | 144 |
| Overall | 262 | 2,626 | 216 | 2,447 |

**Table S6** Raw data used for discontinuation due to LoE outcome

| Trial | Memantine | | Placebo | |
| --- | --- | --- | --- | --- |
|  | Event | Total | Event | Total |
| Ashford et al. 2011 | 0 | 7 | 0 | 6 |
| Bakchine et al. 2008 | 4 | 318 | 2 | 152 |
| Dysken et al. 2014 | 0 | 155 | 0 | 152 |
| Grossberg et al. 2013 | 3 | 342 | 8 | 335 |
| Lorenzi et al. 2011 | 0 | 7 | 0 | 8 |
| Peskind et al. 2006 | 0 | 201 | 5 | 202 |
| Porsteinsson et al. 2008 | 1 | 217 | 1 | 216 |
| Saxton et al. 2012 | 0 | 136 | 0 | 129 |
| Tariot et al. 2004 | 1 | 203 | 3 | 201 |
| Van Dyck et al. 2007 | 0 | 178 | 3 | 172 |
| Wilkinson et al. 2012 | 1 | 134 | 3 | 144 |
| Overall | 10 | 1,898 | 25 | 1,717 |

**Table S7** Raw data analysed for efficacy on cognitive function outcome

| Trial | Scale | SMD | SEM | N |
| --- | --- | --- | --- | --- |
| Ashford et al. 2011 | ADAS-Cog | 0.56 | 0.66 | 10 |
| Bakchine et al. 2008 | ADAS-Cog | 0.13 | 0.11 | 403 |
| Dysken et al. 2014 | ADAS-Cog | 0.17 | 0.12 | 282 |
| Fox et al. 2012 | MMSE | 0.21 | 0.16 | 149 |
| Grossberg et al. 2013 | SIB | 0.21 | 0.08 | 659 |
| Herrmann et al. 2013 | SIB | -0.05 | 0.11 | 324 |
| Howard et al. 2012a | MMSE | 0.35 | 0.2 | 105 |
| Howard et al. 2012b | MMSE | 0.05 | 0.19 | 112 |
| Lorenzi et al. 2011 | MMSE | -0.79 | 0.54 | 15 |
| Lundbeck study 10116 | MMSE | 0.13 | 0.13 | 236 |
| Peskind et al. 2006 | ADAS-Cog | 0.24 | 0.1 | 393 |
| Porsteinsson et al. 2008 | ADAS-Cog | -0.04 | 0.1 | 427 |
| Reisberg et al. 2003 | MMSE | 0.25 | 0.13 | 248 |
| Tariot et al. 2004 | SIB | 0.36 | 0.1 | 394 |
| Van Dyck et al. 2007 | SIB | 0.04 | 0.11 | 335 |
| Wang et al. 2013 | ADAS-Cog | 0.61 | 0.44 | 22 |
| Wilkinson et al. 2012 | MMSE | 0.07 | 0.13 | 222 |

SMD, standardized mean difference; SEM, standard error of the mean; N, number of patients included in the analysis.

**Table S8** Raw data analysed for efficacy on global change outcome

| Trial | Scale | SMD | SEM | N |
| --- | --- | --- | --- | --- |
| Bakchine et al. 2008 | CIBIC-Plus | 0.06 | 0.11 | 403 |
| Fox et al. 2012 | CGI | 0.19 | 0.16 | 149 |
| Grossberg et al. 2013 | CIBIC-Plus | 0.25 | 0.08 | 661 |
| Herrmann et al. 2013 | CIBIC-Plus | -0.04 | 0.12 | 175 |
| Kitamura et al. 2011a | CIBIC-Plus | 0.01 | 0.17 | 161 |
| Kitamura et al. 2011b | CIBIC-Plus | 0.16 | 0.17 | 154 |
| Nakamura et al. 2011 | CIBIC-Plus | 0.16 | 0.10 | 425 |
| Peskind et al. 2006 | CIBIC-Plus | 0.32 | 0.10 | 393 |
| Porsteinsson et al. 2008 | CIBIC-Plus | 0.04 | 0.10 | 427 |
| Reisberg et al. 2003 | CIBIC-Plus | 0.27 | 0.13 | 236 |
| Saxton et al. 2012 | CGI | 0.27 | 0.13 | 257 |
| Tariot et al. 2004 | CIBIC-Plus | 0.24 | 0.10 | 394 |
| Van Dyck et al. 2007 | CIBIC-Plus | 0.02 | 0.11 | 334 |

**Table S9** Raw data analysed for efficacy neuropsychiatric symptoms outcome

| Trial | Scale | SMD | SEM | N |
| --- | --- | --- | --- | --- |
| Bakchine et al. 2008 | NPI | 0.13 | 0.11 | 401 |
| Dysken et al. 2014 | NPI | 0.03 | 0.12 | 282 |
| Fox et al. 2012 | NPI | 0.62 | 0.18 | 137 |
| Grossberg et al. 2013 | NPI | 0.20 | 0.08 | 639 |
| Herrmann et al. 2013 | NPI | 0.08 | 0.11 | 324 |
| Howard et al. 2012a | NPI | 0.36 | 0.20 | 105 |
| Howard et al. 2012b | NPI | 0.30 | 0.19 | 112 |
| Kitamura et al. 2011a | NPI | -0.02 | 0.17 | 161 |
| Kitamura et al. 2011b | NPI | 0.14 | 0.17 | 154 |
| Lundbeck study 10116 | NPI | 0.28 | 0.13 | 236 |
| Nakamura et al. 2011 | NPI | 0.25 | 0.10 | 425 |
| Peskind et al. 2006 | NPI | 0.21 | 0.10 | 381 |
| Porsteinsson et al. 2008 | NPI | -0.02 | 0.10 | 421 |
| Reisberg et al. 2003 | NPI | 0.19 | 0.13 | 239 |
| Tariot et al. 2004 | NPI | 0.28 | 0.10 | 382 |
| Van Dyck et al. 2007 | NPI | 0.01 | 0.11 | 315 |
| Wang et al. 2013 | NPI | 0.09 | 0.43 | 22 |
| Wilkinson et al. 2012 | NPI | 0.04 | 0.12 | 275 |

SMD, standardized mean difference; SEM, standard error of the mean; N, number of patients included in the analysis.

**Table S10** Raw data analysed for efficacy on functional ability outcome

| Trial | Scale | SMD | SEM | N |
| --- | --- | --- | --- | --- |
| Bakchine et al. 2008 | ADCS-ADL_23_ | -0.01 | 0.11 | 401 |
| Dysken et al. 2014 | ADCS-ADL_23_ | 0.15 | 0.12 | 282 |
| Grossberg et al. 2013 | ADCS-ADL_19_ | 0.08 | 0.08 | 659 |
| Hermann et al. 2013 | ADCS-ADL_19_ | -0.23 | 0.12 | 276 |
| Kitamura et al. 2011a | ADCS-ADL | -0.01 | 0.17 | 261 |
| Kitamura et al. 2011b | ADCS-ADL | 0.00 | 0.17 | 154 |
| Lundbeck study 10116 | ADCS-ADL_19_ | 0.09 | 0.13 | 236 |
| Peskind et al. 2006 | ADCS-ADL_23_ | 0.01 | 0.10 | 393 |
| Porsteinsson et al. 2008 | ADCS-ADL_23_ | -0.01 | 0.10 | 427 |
| Reisberg et al. 2003 | ADCS-ADL_19_ | 0.32 | 0.13 | 247 |
| Tariot et al. 2004 | ADCS-ADL_19_ | 0.20 | 0.10 | 395 |
| Van Dyck et al. 2007 | ADCS-ADL_19_ | 0.09 | 0.11 | 336 |

SMD, standardized mean difference; SEM, standard error of the mean; N, number of patients included in the analysis.

**Table S11** Raw data used for proportion patients with AEs outcome

| Trial | Memantine | | Placebo | |
| --- | --- | --- | --- | --- |
|  | Event | Total | Event | Total |
| Bakchine et al. 2008 | 178 | 318 | 80 | 152 |
| Dysken et al. 2014 | 97 | 155 | 89 | 152 |
| Grossberg et al. 2013 | 214 | 342 | 214 | 335 |
| Herrmann et al. 2013 | 138 | 182 | 136 | 187 |
| Reisberg et al. 2003 | 106 | 126 | 109 | 126 |
| Van Dyck et al. 2007 | 131 | 178 | 125 | 172 |
| Overall | 864 | 1,301 | 753 | 1,124 |

**Table S12** Raw data used for proportion patients with SAE outcome

| Trial | Memantine | | Placebo | |
| --- | --- | --- | --- | --- |
|  | Event | Total | Event | Total |
| Bakchine et al. 2008 | 32 | 318 | 9 | 152 |
| Dysken et al. 2014 | 84 | 155 | 95 | 152 |
| Grossberg et al. 2013 | 28 | 342 | 21 | 335 |
| Lundbeck study 10116 | 4 | 128 | 4 | 130 |
| Peskind et al. 2006 | 20 | 201 | 20 | 202 |
| Porsteinsson et al. 2008 | 27 | 217 | 30 | 216 |
| Reisberg et al. 2003 | 16 | 126 | 23 | 126 |
| Saxton et al. 2012 | 4 | 136 | 13 | 129 |
| Van Dyck et al. 2007 | 26 | 178 | 29 | 172 |
| Wilkinson et al. 2012 | 5 | 134 | 6 | 144 |
| Overall | 246 | 1,935 | 250 | 1,758 |

**Table S13** Raw data used for mortality outcome

| Trial | Memantine | | Placebo | |
| --- | --- | --- | --- | --- |
|  | Event | Total | Event | Total |
| Ashford et al. 2011 | 0 | 7 | 0 | 6 |
| Bakchine et al. 2008 | 5 | 318 | 1 | 152 |
| Dysken et al. 2014 | 39 | 155 | 31 | 152 |
| Fox et al. 2012 | 5 | 74 | 7 | 79 |
| Grossberg et al. 2013 | 4 | 342 | 5 | 335 |
| Herrmann et al. 2013 | 1 | 182 | 1 | 187 |
| Howard et al. 2012a | 10 | 76 | 9 | 73 |
| Howard et al. 2012b | 7 | 73 | 13 | 73 |
| Lorenzi et al. 2011 | 0 | 7 | 0 | 8 |
| Lundbeck study 10116 | 3 | 128 | 1 | 130 |
| Peskind et al. 2006 | 1 | 201 | 1 | 202 |
| Porsteinsson et al. 2008 | 3 | 217 | 2 | 216 |
| Reisberg et al. 2003 | 2 | 126 | 5 | 126 |
| Saxton et al. 2012 | 0 | 136 | 1 | 129 |
| Van Dyck et al. 2007 | 5 | 178 | 3 | 172 |
| Overall | 85 | 2,220 | 80 | 2,040 |
