# Supplementary material for: Predictors of discontinuation, efficacy, and safety of memantine treatment for Alzheimer’s disease: meta-analysis and meta-regression of 18 randomized clinical trials involving 5004 patients
Source: BMC Geriatr. 2018 Jul 24;18:168. doi: 10.1186/s12877-018-0857-5 (PMC6057050; doi:10.1186/s12877-018-0857-5)
Supplement: Supplementary file 3 — Characteristics of included clinical trials. We provide the references of included studies (Table S14) and their study-, intervention-, and patient-related characteristics (Table S15 and Table S16). (DOCX 32 kb) [file 12877_2018_857_MOESM3_ESM.docx]

**Table S14** Randomized placebo-controlled clinical trials included in the primary analysis

| Ashford JW, Adamson M, Beale T, et al. MR spectroscopy for assessment of memantine treatment in mild to moderate Alzheimer dementia. J Alzheimers Dis. 2011;26(Suppl 3):331-6.  Bakchine S, Loft H. Memantine treatment in patients with mild to moderate Alzheimer’s disease: results of a randomised, double-blind, placebo-controlled 6-month study. J Alzheimers Dis. 2008;13(1):97-107.  Dysken MW, Guarino PD, Vertrees JE, et al. Effect of vitamin E and memantine on functional decline in Alzheimer disease: the TEAM-AD VA cooperative randomized trial. JAMMA;311(1):33-44.  Fox C, Crugel M, Maidment I, et al. Efficacy of memantine for agitation in Alzheimer’s dementia: A randomised double-blind placebo controlled trial. PLoS One. 2012;7(5):e35185.  Grossberg GT, Manes F, Allegri RF, et al. The safety, tolerability, and efficacy of once-daily memantine (28 mg): A multinational, randomized, double-blind, placebo-controlled trial in patients with moderate-to-severe alzheimer’s disease taking cholinesterase inhibitors. CNS Drugs. 2013; 27(6):469-78.  Herrmann N, Gauthier S, Boneva N, Lemming OM. A randomized, double-blind, placebo-controlled trial of memantine in a behaviorally enriched sample of patients with moderate-to-severe Alzheimer’s disease. Int psychogeriatr. 2013;25(6):919-27.  Howard R, McShane R, Lindesay J, et al. Donepezil and memantine for moderate-to-severe Alzheimer’s disease. N Engl J Med. 2012;366(10):893-903.  Lorenzi M, Beltramello A, Mercuri NB, et al. Effect of Memantine on Resting State Default Mode Network Activity in Alzheimer’s Disease. Drugs & Aging. 2011;28(3):205-17.  Lundbeck. A randomized, double-blind, placebo-controlled evaluation of the efficacy and safety of memantine in patients with dementia of the Alzheimer’s type: Clinical Trial Report Summary– Study 10116. In:Lundbeckwebsite.2006.http://<http://www.lundbeck.com/upload/trials/files/pdf/completed/10116_Final_11Oct2006_CTRS.pdf>. Accessed 1 Feb 2017.  Peskind ER, Potkin SG, Pomara N, et al. Memantine treatment in mild to moderate Alzheimer disease: a 24-week randomized, controlled trial. Am J Geriatr Psychiatry. 2006;14(8):704-15.  Porsteinsson AP, Grossberg GT, Mintzer J, Olin JT. Memantine treatment in patients with mild to moderate Alzheimer’s disease already receiving a cholinesterase inhibitor: a randomized, double-blind, placebo-controlled trial. Curr Alzheimer Res. 2008;5(1):83-9.  Reisberg B, Doody R, Stoffler A, et al. Memantine in moderate-to-severe Alzheimer’s disease. N Engl J Med. 2003; 348(14):1333-41.  Saxton J, Hofbauer RK, Woodward M, et al. Memantine and functional communication in Alzheimer’s disease: Results of a 12-week, international, randomized clinical trial. J Alzheimer’s Dis. 2012;28(1):109-18.  Schmidt R, Ropele S, Pendl B, et al. Longitudinal multimodal imaging in mild to moderate Alzheimer disease: a pilot study with memantine. J Neurol Neurosurg Psychiatry. 2008;79(12):1312-27.  Tariot PN, Farlow MR, Grossberg GT, et al. Memantine treatment in patients with moderate to severe Alzheimer disease already receiving donepezil: a randomized controlled trial. JAMA. 2004;291(3):317-24.  van Dyck CH, Tariot PN, Meyers B, Malca Resnick E. A 24-week randomized, controlled trial of memantine in patients with moderate-to-severe Alzheimer disease. Alzheimer Dis Assoc Disord. 2007;21(2):136-43.  Wang T, Huang Q, Reiman EM, et al. Effects of memantine on clinical ratings, fluorodeoxyglucose positron emission tomography measurements, and cerebrospinal fluid assays in patients with moderate to severe Alzheimer dementia: a 24-week, randomized, clinical trial. J Clin Psychopharmacol. 2013;33(5):636-42.  Wilkinson D, Fox NC, Barkhof F, et al. Memantine and brain atrophy in Alzheimer’s disease: A 1-year randomized controlled trial. J Alzheimers Dis. 2012;29(2):459-69. |
| --- |

**Table S15** Characteristics of included memantine clinical trials in patients with Alzheimer’s disease

| Trial | Multi-site study | Phase lead-in | Placebo lead-in | Diagnostic criteria | AD severity | Intervention | Regimen | Dose  (mg/day) | Dosage | Length (weeks) | N | Age (years) | Women (%) | Baseline cognitive function (%) | Baseline neuropsychiatric symptoms  severity  (%) | Baseline functional ability (%) | Scale cognitive function | Scale global change | Scale neuropsychiatric symptoms | Scale functional capacity | Sponsor |
| --- | --- | --- | --- | --- | --- | --- | --- | --- | --- | --- | --- | --- | --- | --- | --- | --- | --- | --- | --- | --- | --- |
| Ashford et al. 2011 | No | No | No | DSM-IV | mild-moderate | monotherapy | BID | 20 | Fix | 54 | 13 | 76.0 | 38.5 | 33.2 | - | 79.6 | ADAS-Cog | - | - | - | Industry |
| Bakchine et al. 2008 | Yes | No | No | DSM-IV  NINCDS-ADRDA | mild-moderate | monotherapy | BID | 20 | Fix | 24 | 470 | 73.8 | 63.2 | 63.5 | 8.6 | 72.9 | ADAS-cog | CIBIC-Plus | NPI | ADCS-ADL_23_ | Industry |
| Dysken et al. 2014 | Yes | No | No | NINCDS-ADRDA | mild-moderate | combination ChEI | BID | 20 | Fix | 208 | 307 | 79.1 | 2.9 | 72.4 | 5.6 | 73.1 | ADAS-Cog | - | NPI | ADCS-ADL_23_ | Independent |
| Fox et al. 2012 | Yes | No | No | NINCDS-ADRDA | Moderate-severe | monotherapy | BID | 20 | Fix | 12 | 153 | 84.6 | 73.8 | 24.3 | 25.4 | - | MMSE | CGI | NPI | - | Industry |
| Grossberg et al. 2013 | Yes | Yes | Yes | DSM-IV  NINCDS-ADRDA | moderate-severe | combination ChEI | QD | 28 ER | Flexible | 24 | 677 | 76.5 | 72.0 | 35.8 | 11.7 | 66.1 | SIB | CIBIC-Plus | NPI | ADCS-ADL_19_ | Industry |
| Herrmann et al. 2013 | Yes | No | No | NINCDS-ADRDA | moderate-severe | combination ChEI | QD | 20 | Fix | 24 | 369 | 74.9 | 58.3 | 39.5 | 20.9 | 66.3 | SIB | CIBIC-Plus | NPI | ADCS-ADL_19_ | Industry |
| Howard et al. 2012a | Yes | No | No | NINCDS-ADRDA | moderate-severe | monotherapy | QD | 20 | Fix | 52 | 149 | 76.9 | 62.4 | 30.5 | 16.0 | - | MMSE | - | NPI | - | Independent |
| Howard et al. 2012b | Yes | No | No | NINCDS-ADRDA | moderate-severe | combination ChEI | QD | 20 | Fix | 52 | 146 | 77.4 | 68.5 | 30.2 | 14.8 | - | MMSE | - | NPI | - | Independent |
| Kitamura et al. 2011a* | Yes | Yes | Yes | DSM-IV  NINCDS-ADRDA | moderate-severe | monotherapy | QD | 10 | Fix | 24 | 161 | 73.3 | 72.7 | 33.3 | - | - | - | CIBIC-Plus | NPI | - | Industry |
| Kitamura et al. 2011b* | Yes | Yes | Yes | DSM-IV  NINCDS-ADRDA | moderate-severe | monotherapy | QD | 20 | Fix | 24 | 154 | 73.3 | 68.9 | 34.0 | - | - | - | CIBIC-Plus | NPI | - | Industry |
| Lorenzi et al. 2011 | No | No | No | NINCDS-ADRDA | moderate-severe | monotherapy | QD | 20 | Fix | 26 | 15 | 76.5 | 86.7 | 47.5 | - | - | MMSE | - | - | - | Industry |
| Lundbeck study 11016 | Yes | No | No | NINCDS-ADRDA | moderate-severe | monotherapy | BID | 20 | Fix | 16 | 258 | 72.3 | 30.1 | 39.7 | 9.9 | 56.3 | MMSE | - | NPI | ADCS-ADL_19_ | Industry |
| Nakamura et al. 2011* | Yes | Yes | Yes | DSM-IV  NINCDS-ADRDA | moderate-severe | monotherapy | QD | 20 | Fix | 24 | 426 | 74.6 | 64.3 | 32.9 | - | - | - | CIBIC-Plus | NPI | - | Industry |
| Peskind et al. 2006 | Yes | Yes | Yes | NINCDS-ADRDA | mild-moderate | monotherapy | BID | 20 | Fix | 24 | 403 | 77.5 | 58.8 | 61.2 | 8.2 | 72.7 | ADAS-Cog | CIBIC-Plus | NPI | ADCS-ADL_23_ | Industry |
| Porsteinsson et al. 2008 | Yes | Yes | Yes | NINCDS-ADRDA | mild-moderate | combination ChEI | QD | 20 | Fix | 24 | 433 | 75.4 | 52.2 | 60.9 | 8.4 | 70.2 | ADAS-Cog | CIBIC-Plus | NPI | ADCS-ADL_23_ | Industry |
| Reisberg et al. 2003 | Yes | No | No | DSM-IV  NINCDS-ADRDA | moderate-severe | monotherapy | BID | 20 | Fix | 28 | 252 | 76.2 | 67.5 | 26.3 | 14.2 | 50.2 | MMSE | CIBIC-Plus | NPI | ADCS-ADL_19_ | Industry |
| Saxton et al. 2012 | Yes | Yes | Yes | NINCDS-ADRDA | moderate | combination ChEI | BID | 20 | Fix | 12 | 265 | 74.9 | 58.3 | 52.8 | - | - | - | CGI | - | - | Industry |
| Schmidt et al. 2008 | No | No | No | DSM-IV  NINCDS-ADRDA | mild-moderate | monotherapy | BID | 20 | Fix | 52 | 36 | 76.2 | 63.9 | 60.7 | - | - | - | - | - | - | Industry |
| Tariot et al. 2004 | Yes | Yes | Yes | NINCDS-ADRDA | moderate-severe | combination ChEI | BID | 20 | Fix | 24 | 404 | 75.5 | 65.0 | 33.5 | 9.3 | 66.0 | SIB | CIBIC-Plus | NPI | ADCS-ADL_19_ | Industry |
| Van Dyck et al. 2007 | Yes | Yes | Yes | NINCDS-ADRDA | moderate-severe | monotherapy | BID | 20 | Fix | 24 | 350 | 78.2 | 71.4 | 33.8 | 13.1 | 61.7 | SIB | CIBIC-Plus | NPI | ADCS-ADL_19_ | Industry |
| Wang et al. 2013 | No | No | No | DSM-IV  NINCDS-ADRDA | moderate-severe | monotherapy | BID | 20 | Fix | 24 | 26 | 65.2 | 63.6 | 38.4 | 4.4 | - | ADAS-Cog | CIBIC-Plus | NPI | - | Industry |
| Wilkinson et al. 2012 | No | No | No | NINCDS-ADRDA | moderate | combination ChEI | QD | 20 | Fix | 52 | 278 | 74.0 | 57.0 | 56.4 | 9.0 | - | MMSE | - | NPI | - | Industry |

ADAS-Cog, Alzheimer’s Disease Assessment Scale- Cognitive subscale; ADCS-ADL, Alzheimer’s Disease Cooperative Study Activities of Daily Living; BID, twice daily; CGI, Clinical Global Impression; ChEI, Cholinesterase inhibitor; CIBIC-Plus, Clinician Interview-Based Impression on Change-Plus Caregiver Input; DSM-IV, Diagnostic and Statistical Manual of Mental Disorders, ER, extended-release formulation; Fourth Edition; MMSE, Mini-Mental State Examination; N, number of patients; NINCDS-ADRDA, National Institute of Neurological and Communicative Disorders and Stroke and the Alzheimer’s Disease and Related Disorders Association; NPI, Neuropsychiatric Inventory; QD: once daily.

*Comparisons included in the sensitivity analysis

**Table S16** Type of statistical analysis used for efficacy outcomes in memantine clinical trials in patients with Alzheimer’s disease

| Trial | Cognitive function | Global change | Neuropsychiatric symptoms | Functional ability |
| --- | --- | --- | --- | --- |
| Ashford et al. 2011 | non-ITT | - | - | - |
| Bakchine et al. 2008 | non-ITT | non-ITT | non-ITT | non-ITT |
| Dysken et al. 2014 | non-ITT | - | non-ITT | non-ITT |
| Fox et al. 2012 | ITT | ITT | non-ITT | - |
| Grossberg et al. 2013 | ITT | ITT | non-ITT | - |
| Herrmann et al. 2013 | non-ITT | non-ITT | non-ITT | ITT |
| Howard et al. 2012a | non-ITT | - | non-ITT | non-ITT |
| Howard et al. 2012b | non-ITT | - | non-ITT | non-ITT |
| Kitamura et al. 2011a* | - | ITT | ITT | ITT |
| Kitamura et al. 2011b* | - | ITT | ITT | ITT |
| Lorenzi et al. 2011 | ITT | - | - | - |
| Lundbeck study 11016 | non-ITT | - | non-ITT | non-ITT |
| Nakamura et al. 2011* | - | ITT | ITT | - |
| Peskind et al. 2006 | ITT | ITT | non-ITT | ITT |
| Porsteinsson et al. 2008 | ITT | ITT | ITT | ITT |
| Reisberg et al. 2003 | ITT | non-ITT | non-ITT | ITT |
| Saxton et al. 2012 | - | ITT | - | - |
| Schmidt et al. 2008 | - | - | - | - |
| Tariot et al. 2004 | ITT | ITT | non-ITT | ITT |
| Van Dyck et al. 2007 | ITT | ITT | non-ITT | ITT |
| Wang et al. 2013 | non-ITT | - | non-ITT | - |
| Wilkinson et al. 2012 | ITT | - | ITT | - |
| Overall | ITT: 9  Non-ITT: 8 | ITT: 10  Non-ITT: 3 | ITT: 5  Non-ITT: 13 | ITT: 8  Non-ITT: 5 |

ITT, intention-to-treat analysis; non-ITT, non-intention to treat analysis

*Comparisons included in the sensitivity analysis
