# Supplementary material for: Predictors of discontinuation, efficacy, and safety of memantine treatment for Alzheimer’s disease: meta-analysis and meta-regression of 18 randomized clinical trials involving 5004 patients
Source: BMC Geriatr. 2018 Jul 24;18:168. doi: 10.1186/s12877-018-0857-5 (PMC6057050; doi:10.1186/s12877-018-0857-5)
Supplement: Supplementary file 4 — Risk of bias of included clinical trials. We provide the risk of bias of included studies on different domains using the Cochrane Collaboration tool (Figure S2 and Figure. S3) and the high risk of bias by study outcomes (Table S17). (DOCX 73 kb) [file 12877_2018_857_MOESM4_ESM.docx]

**Figure S1** Risk of bias memantine-placebo comparison in included clinical trials

**
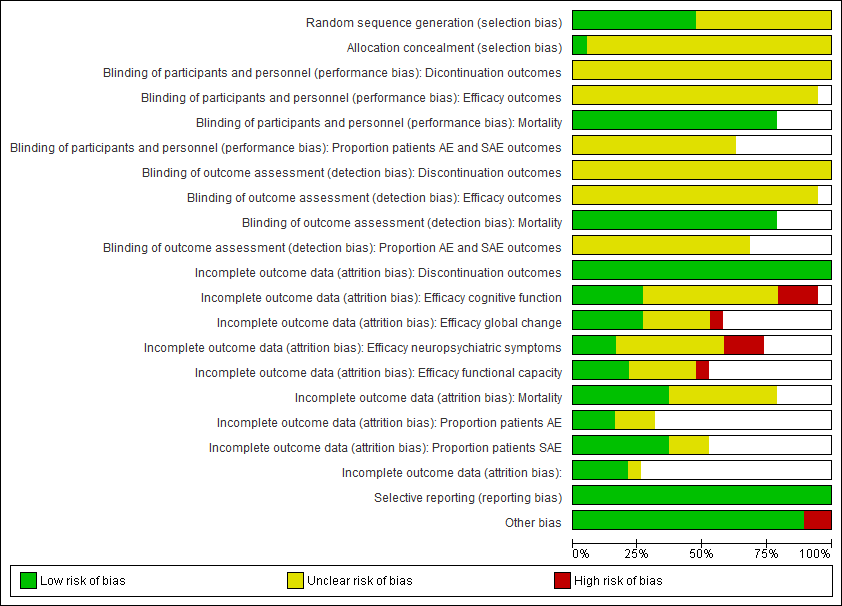
**

**Figure S2** Risk of bias memantine-placebo comparison in included clinical trials
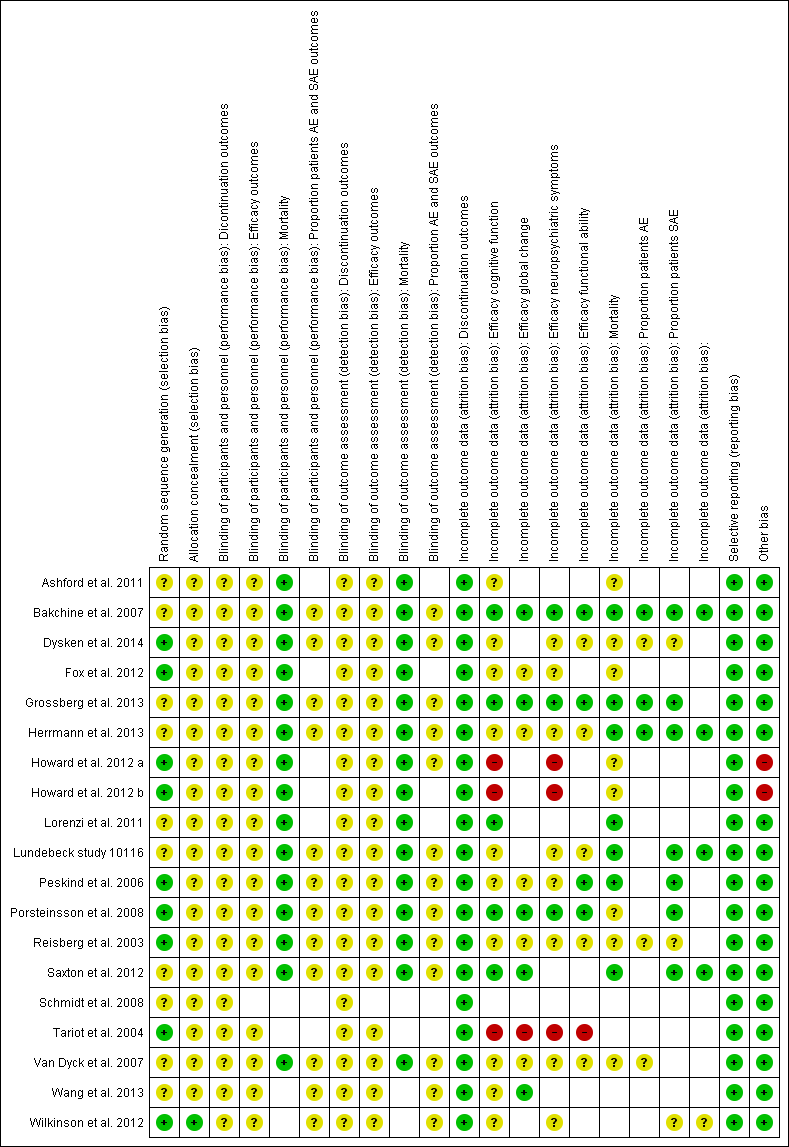


**Table S17** High risk of bias of included clinical trials of memantine in patients with Alzheimer’s disease

| Trial | Discontinuation outcomes | Cognitive function | Global change | Neuropsychiatric symptoms | Functional ability | Proportion patients AE | Proportion patients SAE | Mortality |
| --- | --- | --- | --- | --- | --- | --- | --- | --- |
| Ashford et al. 2011 | No | No | - | - | - | - | - | No |
| Bakchine et al. 2008 | No | No | No | No | No | No | No | No |
| Dysken et al. 2014 | No | No | - | No | No | No | No | No |
| Fox et al. 2012 | No | No | No | No | - | - | - | No |
| Grossberg et al. 2013 | No | No | No | No | No | No | No | No |
| Herrmann et al. 2013 | No | No | No | No | No | No | - | No |
| Howard et al. 2012a | No | Yes | - | Yes | - | - | - | No |
| Howard et al. 2012b | No | Yes | - | Yes | - | - | - | No |
| Lorenzi et al. 2011 | No | No | - | - | - | - | - | No |
| Lundbeck study 10116 | No | No | - | No | No | - | No | No |
| Peskind et al. 2006 | No | No | No | No | No | - | No | No |
| Porsteinsson et al. 2008 | No | No | No | No | No | - | No | No |
| Reisberg et al. 2003 | No | No | No | No | No | No | No | No |
| Saxton et al. 2012 | No | - | No | - | - | - | No | No |
| Schmidt et al. 2008 | No | - | - | - | - | - | - | - |
| Tariot et al. 2004 | No | Yes | Yes | Yes | Yes | - | - | - |
| Van Dyck et al. 2007 | No | No | No | No | No | No | No | No |
| Wang et al. 2013 | No | No | - | No | - | - | - | - |
| Wilkinson et al. 2012 | No | No | - | No | - | - | No | - |
| Overall | Yes: 0  No: 19 | Yes: 3  No: 14 | Yes: 1  No: 9 | Yes: 3  No: 12 | Yes: 1  No: 9 | Yes: 0  No: 8 | Yes: 0  No: 10 | Yes: 0  No: 15 |
