# Supplementary material for: Predictors of discontinuation, efficacy, and safety of memantine treatment for Alzheimer’s disease: meta-analysis and meta-regression of 18 randomized clinical trials involving 5004 patients
Source: BMC Geriatr. 2018 Jul 24;18:168. doi: 10.1186/s12877-018-0857-5 (PMC6057050; doi:10.1186/s12877-018-0857-5)
Supplement: Supplementary file 5 — Forest plot of secondary outcomes and additional results. We provide the forest plot of secondary outcomes (Figure S3, Figure S4, Figure S5, Figure S6, Figure S7, Figure S8 and Figure S9), the results of meta-regression of some secondary outcomes not provided in the manuscript (Table S18) and the results of the meta-analysis of study outcomes by type of intervention (monotherapy vs. combination with ChEI) (Table S19). (DOCX 249 kb) [file 12877_2018_857_MOESM5_ESM.docx]

**Figure S3** Forest plot of the effect of memantine on discontinuation due to LoE.

**
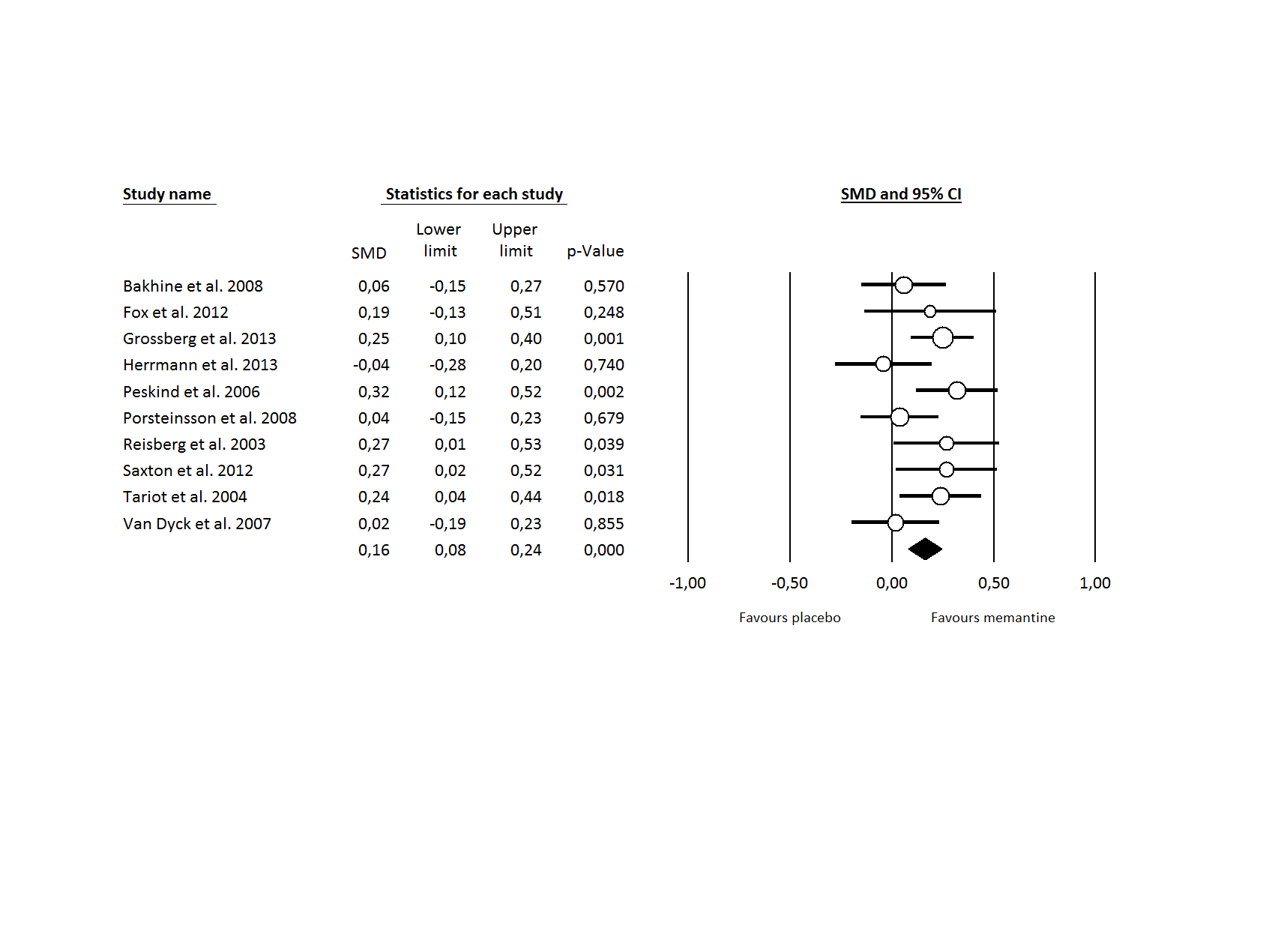
**

**Figure S4** Forest plot of the effect of memantine on efficacy on global change.


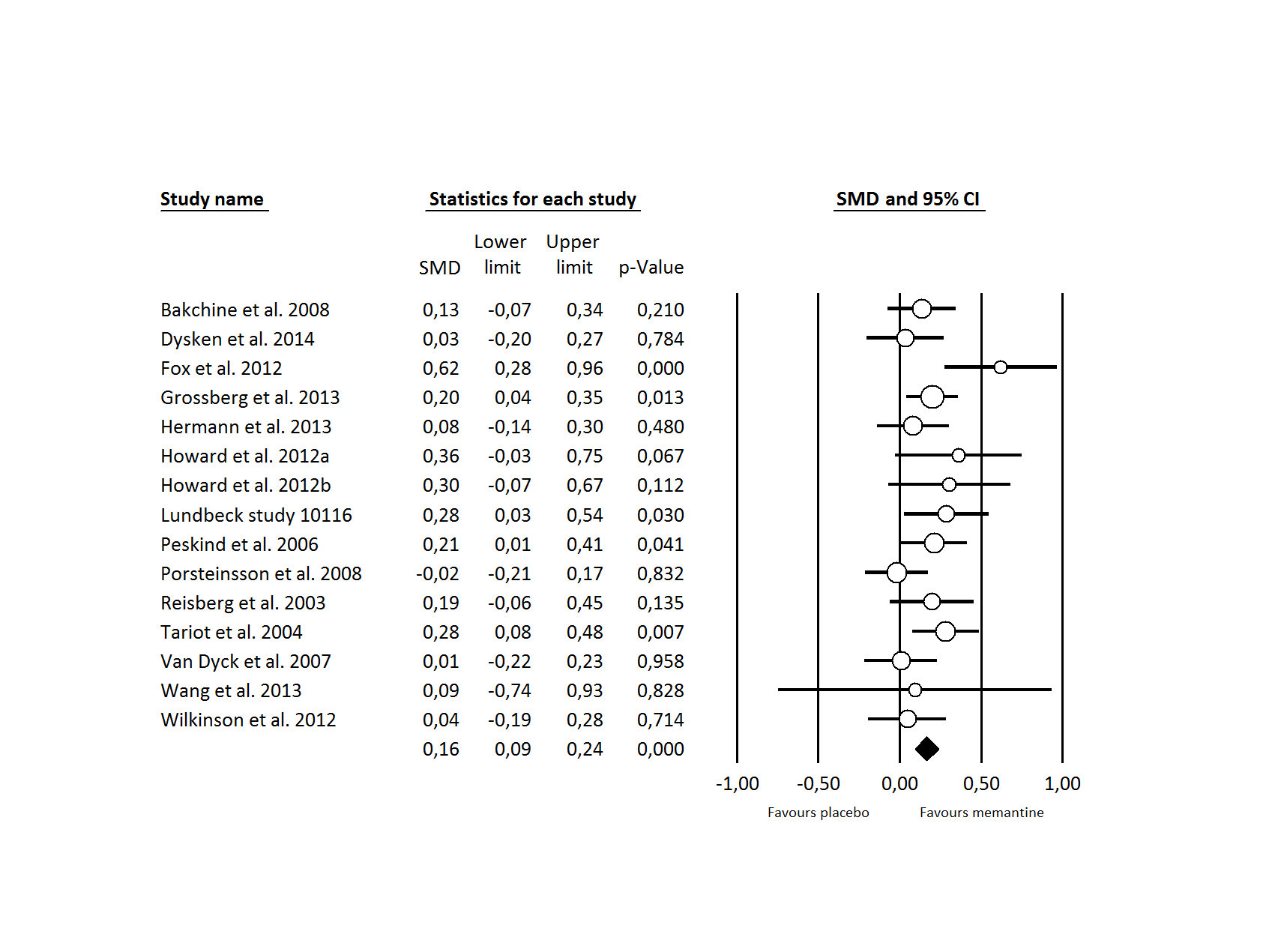


**Figure S5** Forest plot of the effect of memantine on efficacy on neuropsychiatric symptoms.


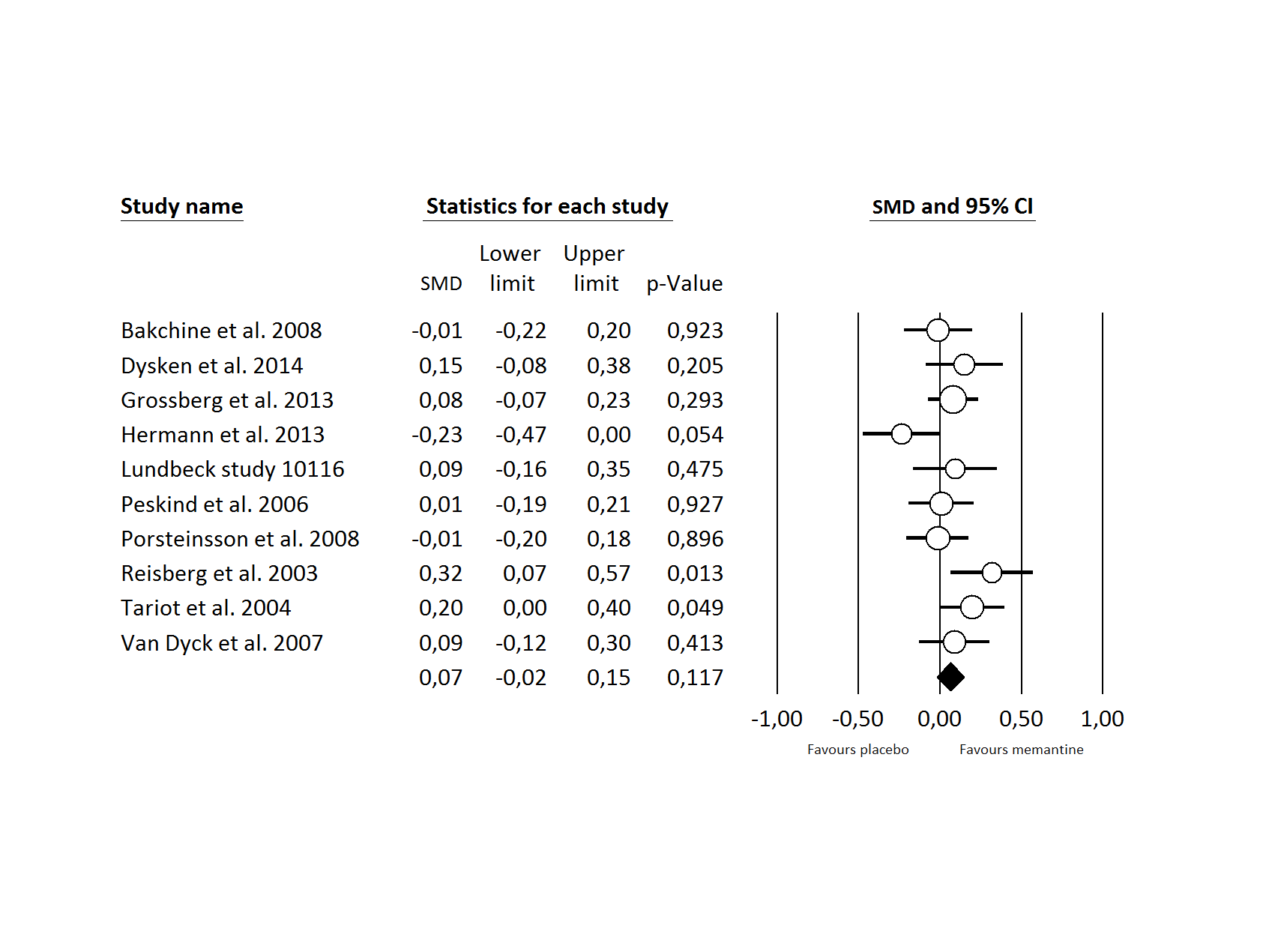


**Figure S6** Forest plot of the effect of memantine on efficacy on functional ability.

**Figure S7** Forest plot of the effect of memantine on proportion patients with AE.

**Figure S8** Forest plot of the effect of memantine on proportion patients with SAE.

**Figure S9** Forest plot of the effect of memantine on mortality.

**Table S18** Meta-regression analysis of study outcomes

|  | Discontinuation due to LoE | | | | Proportion patients AE | | | Mortality | | |
| --- | --- | --- | --- | --- | --- | --- | --- | --- | --- | --- |
|  | N | | Constant  (95%CI)  Log OR  (95%CI) | R^2^ | N | Constant  (95%CI)  Log OR  (95%CI) | R^2^ | N | Constant  (95%CI)  Log OR  (95%CI) | R^2^ |
| Study site |  | |  |  |  |  |  |  |  |  |
| Single site (ref.) | - | | - | - | - | - | - | - | - | - |
| Multi-site |  | | - |  |  | - |  |  | - |  |
| Lead-in period |  | |  |  |  |  |  |  |  |  |
| No (ref.) | 7 | | -0.404  (-1.774, 0.962) | 0 | 6 | 0.114  (-0.122, 0.349) | 0 | 13 | 0.021  (-0.352, 0.394) | 0 |
| Yes |  | | -0.762  (-2.424, 0.901) |  |  | -0.139  (-0.490, 0.213) |  |  | 0.046  (-0.832, 0.923) |  |
| Placebo lead-in period |  | |  |  |  |  |  |  |  |  |
| No (ref.) | 7 | | -0.404  (-1.774, 0.962) | 0 | 6 | 0.114  (-0.122, 0.349) | 0 | 13 | 0.021  (-0.352, 0.394) | 0 |
| Yes |  | | -0.762  (-2.424, 0.901) |  |  | -0.139  (-0.490, 0.213) |  |  | 0.046  (-0.832, 0.923) |  |
| Statistical analysis |  | |  |  |  |  |  |  |  |  |
| ITT (ref.) | - | | - | - | - | - | - | - | - | - |
| Non-ITT |  | | - |  |  | - |  |  | - |  |
| Intervention |  | |  |  |  |  |  |  |  |  |
| Monotherapy (ref.) | 7 | | -0.921  (-2.240, 0.398) | 0 | 6 | 0.055  (-0.221, 0.330) | 0 | 13 | 0.055  (-0.508, 0.618) | 0 |
| Combination ChEI |  | | 0.003  (-1.630, 1.637) |  |  | -0.005  (-0.361, 0.352) |  |  | -0.040  (-0.744, 0.663) |  |
| Dose |  | |  |  |  |  |  |  |  |  |
| 20 mg/day (ref.) | 7 | | -0.868  (-1.826, 0.089) | 0 | 6 | 0.101  (-0.110, 0.311) | 0 | 13 | 0.048  (-0.301, 0.397) | 0 |
| 28 mg/day |  | | -0.149  (-1.792, 1.495) |  |  | -0.157  (-0.534, 0.220) |  |  | -0.295  (-1.664, 1.073) |  |
| Dosage |  | |  |  |  |  |  |  |  |  |
| Fixed (ref.) | 7 | | -0.868  (-1.826, 0.089) | 0 | 6 | 0.101  (-0.110, 0.311) | 0 | 13 | 0.048  (-0.301, 0.397) | 0 |
| Flexible |  | | -0.149  (-1.792, 1.495) |  |  | -0.157  (-0.534, 0.220) |  |  | -0.295  (-1.664, 1.073) |  |
| Regimen |  | |  |  |  |  |  |  |  |  |
| QD (ref.) | 7 | | -0.874  (-1.938, 0.191) | 0 | 6 | 0.011  (-0.249, 0.271) | 0 | 13 | -0.215  (-0.781, 0.351) | 0 |
| BID |  | | -0.097  (-1.657, 1.463) |  |  | 0.074  (-0.278, 0.425) |  |  | 0.379  (-0.326, 1.084) |  |
| Length (weeks) |  | |  |  |  |  |  |  |  |  |
| Intercept | 7 | | -0.785  (-3.268, 1.699) | 0 | 6 | 0.014  (-0.208, 0.237) | 0 | 13 | -0.185  (-0.709, 0.340) | 0 |
|  |  | | -0.005  (-0.091, 0.082) |  |  | 0.001  (-0.002, 0.003) |  |  | 0.002  (-0.002, 0.006) |  |
| Age (years) |  | |  |  |  |  |  |  |  |  |
| Intercept | 7 | | 27.546  (-14.359, 69.451) | 0 | 6 | 0.592  (-6.878, 8.063) | 0 | 13 | 1.751  (-8.999, 12.500) | 0 |
|  |  | | -0.376  (-0.930, 0.178) |  |  | -0.007  (-0.105, 0.091) |  |  | -0.022  (-0.159, 0.115) |  |
| Women (%) |  | |  |  |  |  |  |  |  |  |
| Intercept | 7 | | 0.847  (-6.881, 8.575) | 0 | 6 | 0.210  (-0.260, 0.680) | 0 | 13 | 0.315  (-0.243, 0.872) | 0 |
|  |  | | -0.027  (-0.145, 0.091) |  |  | -0.003  (-0.010, 0.005) |  |  | -0.007  (-0.018, 0.004) |  |
| AD baseline severity | | | | | | | | | | |
| Mild-moderate (ref.) | | 7 | -0.513  (-1.814, 0.787) | 0 | 6 | 0.097  (-0.175, 0.370) | 0 | 13 | 0.305  (-0.187, 0.797) | 0 |
| Moderate-severe | |  | -0.631  (-2.254, 0.992) |  |  | -0.078  (-0.433, 0.277) |  |  | -0.522  (-1.198, 0.155) |  |
| Baseline cognitive function (mean) | | | | | | | | | | |
| Intercept | | 7 | -2.022  (-4.940, 0.897) | 0 | 6 | -0.257  (-0.821, 0.307) | 0 | 13 | -0.630  (-1.532, 0.272) | 0 |
|  | |  | 0.023  (-0.036, 0.083) |  |  | 0.006  (-0.005, 0.017) |  |  | 0.013  (-0.004, 0.030) |  |
| Baseline neuropsychiatric symptoms severity (mean) | | | | | | | | | | |
| Intercept | 7 | | 0.724  (-4.136, 5.558) | 0 | 6 | 0.084  (-0.412, 0.580) | 0 | 12 | 0.486  (-0.236, 1.209) | 0 |
|  |  | | -0.163  (-0.641, 0.314) |  |  | -0.003  (-0.042, 0.037) |  |  | -0.039  (-0.096, 0.017) |  |
| Baseline functional ability (mean) | | | |  |  |  |  |  |  |  |
| Intercept | 7 | | -7.632  (-23.425, 8.161) | 0 | 6 | -0.885  (-2.887, 1.117) | 0 | 9 | -1.653  (-6.000, 2.694) | 0 |
|  |  | | 0.099  (-0.133, 0.330) |  |  | 0.014  (-0.016, 0.044) |  |  | 0.027  (-0.036, 0.089) |  |
| Type of scale | | |  |  |  |  |  |  |  |  |
| Intercept (ref.) | - | | - | - | - | - | - | - | - | - |
|  |  | | - |  |  | - |  |  | - |  |
| Sponsor | | |  |  |  |  |  |  |  |  |
| Independent (ref.) | - | | - | - | 6 | 0.169  (-0.290, 0.627) | 0 | 13 | 0.051  (-0.372, 0.474) | 0 |
| Industry |  | | - |  |  | -0.137  (-0.633, 0.359) |  |  | -0.061  (-0.763, 0.641) |  |

^a^Type of scale used to evaluate the efficacy. Cognitive function ADAS-Cog, MMSE or SIB, global change CIBIC-Plus or CGI, neuropsychiatric symptoms NPI, functional capacity ADCS-ADL

*Statistically significant effect (p-value ≤0.05) ** statistically significant effect (p-value ≤0.01)

AD, Alzheimer’s disease; AE, adverse events; CI, confidence interval; Diff SMD, difference of standardized mean difference; ITT, intention to treat analysis; LoE, lack of efficacy; Log OR, Log odd ratio; N, number of memantine-placebo comparisons; NA, not applicable; PP, per-protocol analysis; R^2^ , proportion of variance explained by the covariate; SAE, serious adverse events.

**Table S19** Effect of memantine on discontinuation, efficacy and safety outcomes in patients with Alzheimer’s disease

|  | Monotherapy | | | | | Combination ChEI | | | |  |
| --- | --- | --- | --- | --- | --- | --- | --- | --- | --- | --- |
|  | N^*^ | OR | | (95%CI) | *I^2^* (%) | N^*^ | OR | (95%CI) | *I^2^* (%) | Test subgroups differences  (p-value) |
| All-cause discontinuation | 10 | 1.06 | (0.82, 1.35) | | 16.7 | 8 | 0.90 | (0.72, 1.13) | 16.7 | 0.358 |
| Discontinuation due to AE | 7 | 1.28 | (0.85, 1.92) | | 45.2 | 7 | 1.11 | (0.77, 1.61) | 31.7 | 0.623 |
| Discontinuation due to LoE | 3 | 0.40 | (0.11, 1.49) | | 21.1 | 4 | 0.40 | (0.15, 1.05) | 0 | 0.997 |
|  | N | SMD | (95%CI) | | *I^2^* (%) | N | SMD | (95%CI) | *I^2^* (%) | Test subgroups differences  (p-value) |
| Cognitive function | 10 | 0.18 | (0.07, 0.29) | | 0 | 7 | 0.13 | (0.02, 0.23) | 52.5 | 0.491 |
| Global change | 5 | 0.17 | (0.04, 0.30) | | 30.3 | 5 | 0.16 | (0.04, 0.28) | 42.8 | 0.923 |
| Neuropsychiatric symptoms | 8 | 0.21 | (0.10, 0.32) | | 31.5 | 7 | 0.12 | (0.02, 0.22) | 19.3 | 0.231 |
| Functional capacity | 5 | 0.09 | (-0.04, 0.22) | | 0 | 5 | 0.05 | (-0.07, 0.17) | 55.4 | 0.626 |
|  | N | OR | (95%CI) | | *I^2^* (%) | N | OR | (95%CI) | *I^2^* (%) | Test subgroups differences  (p-value) |
| Proportion patients AE | 3 | 1.06 | (0.80, 1.39) | | 0 | 3 | 1.05 | (0.84, 1.32) | 0 | 0.978 |
| Proportion patients SAE | 5 | 0.97 | (0.67, 1.41) | | 0 | 5 | 0.82 | (0.58, 1.17) | 40.1 | 0.534 |
| Mortality | 7 | 1.06 | (0.60, 1.86) | | 0 | 6 | 1.02 | (0.67, 1.55) | 0 | 0.911 |

AE, adverse events; ChEI, cholinesterase inhibitor; CI, confidence interval; *I^2^*, heterogeneity; LoE, lack of efficacy; N, number of memantine-placebo comparisons; OR, odds ratio; SAE, severe adverse events.

^*^ One study included had a factorial design
