# Supplementary material for: Predictors of discontinuation, efficacy, and safety of memantine treatment for Alzheimer’s disease: meta-analysis and meta-regression of 18 randomized clinical trials involving 5004 patients
Source: BMC Geriatr. 2018 Jul 24;18:168. doi: 10.1186/s12877-018-0857-5 (PMC6057050; doi:10.1186/s12877-018-0857-5)
Supplement: Supplementary file 6 — Sensitivity analyses and publication bias. We provide the results of the two sensitivity analyses: by excluding studies with high risk of bias (Table S20) and by including the results of Nakamura et al. 2014 pooled analysis (Table S21); and the funnel plots of study outcomes (Figure S10). (DOCX 291 kb) [file 12877_2018_857_MOESM6_ESM.docx]

**Table S20** Sensitivity analysis by excluding studies with high risk of bias

|  | OR | (95%CI) | *I^2^* (%) | Test subgroups differences  (p-value) |
| --- | --- | --- | --- | --- |
| All-cause discontinuation | 0.97 | (0.82, 1.14) | 14.9 | 0.358 |
| Discontinuation due to AE | 1.18 | (0.89, 1.56) | 34.4 | 0.623 |
| Discontinuation due to LoE | 0.40 | (0.18, 0.87) | 0 | 0.997 |
|  | SMD | (95%CI) | *I^2^* (%) | Test subgroups differences  (p-value) |
| Cognitive function | 0.12 | (0.05, 0.20) | 11.8 | 0.272 |
| Global change | 0.15 | (0.06, 0.25) | 34.1 | 0.755 |
| Neuropsychiatric symptoms | 0.14 | (0.02, 0.26) | 30.0 | 0.119 |
| Functional capacity | 0.05 | (-0.04, 0.14) | 32.8 | 0.375 |
|  | OR | (95%CI) | *I^2^* (%) | Test subgroups differences  (p-value) |
| Proportion patients AE | 1.05 | (0.88, 1.25) | 0 | 0.978 |
| Proportion patients SAE | 0.89 | (0.69, 1.15) | 18.3 | 0.534 |
| Mortality | 1.03 | (0.74, 1.44) | 0 | 0.911 |

AE, adverse events; LoE, Lack of efficacy; CI, confidence interval; *I^2^* , heterogeneity; OR, Odds ratio; SAE, severe adverse events; SMD, standardized mean difference.

**Table S21** Sensitivity analysis by including Nakamura et al. 2014 pooled analysis

|  | OR | (95%CI) | *I^2^* (%) | Test subgroups differences  (p-value) |
| --- | --- | --- | --- | --- |
| All-cause discontinuation | 0.94 | (0.81, 1.10) | 10.7 | 0.614 |
| Discontinuation due to AE | 1.17 | (0.91, 1.51) | 29.7 | 0.702 |
| Discontinuation due to LoE | 0.40 | (0.18, 0.87) | 0 | 0.997 |
|  | SMD | (95%CI) | *I^2^* (%) | Test subgroups differences  (p-value) |
| Cognitive function | 0.15 | (0.08, 0.22) | 24.3 | 0.491 |
| Global change | 0.16 | (0.09, 0.22) | 11.6 | 0.907 |
| Neuropsychiatric symptoms | 0.16 | (0.09, 0.23) | 19.8 | 0.254 |
| Functional capacity | 0.06 | (-0.02, 0.14) | 22.4 | 0.758 |
|  | OR | (95%CI) | *I^2^* (%) | Test subgroups differences  (p-value) |
| Proportion patients AE | 1.05 | (0.88, 1.25) | 0 | 0.978 |
| Proportion patients SAE | 0.89 | (0.69, 1.15) | 18.3 | 0.534 |
| Mortality | 1.03 | (0.74, 1.44) | 0 | 0.911 |

AE, adverse events; LoE, Lack of efficacy; CI, confidence interval; *I^2^* , heterogeneity; OR, Odds ratio; SAE, severe adverse events; SMD, standardized mean difference.

| 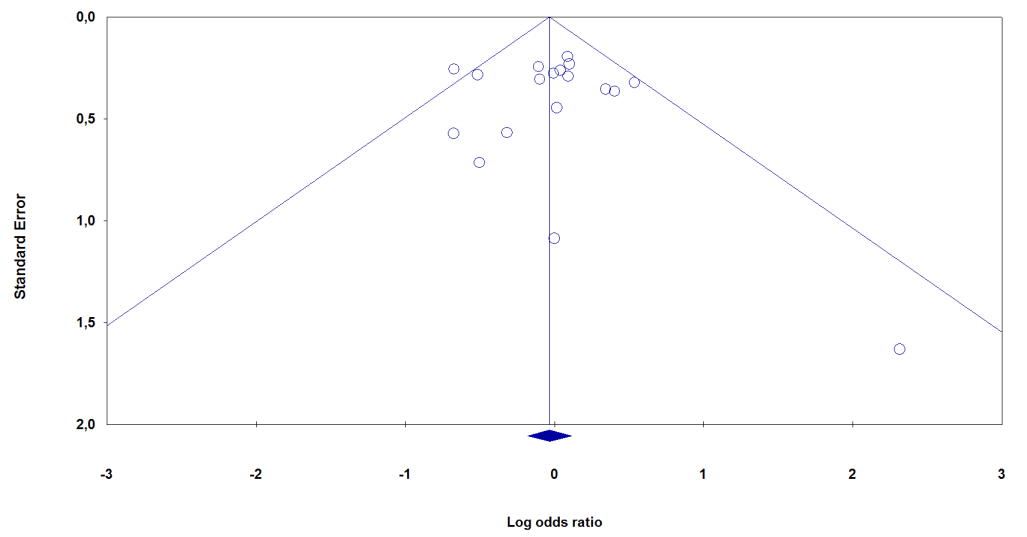  A | 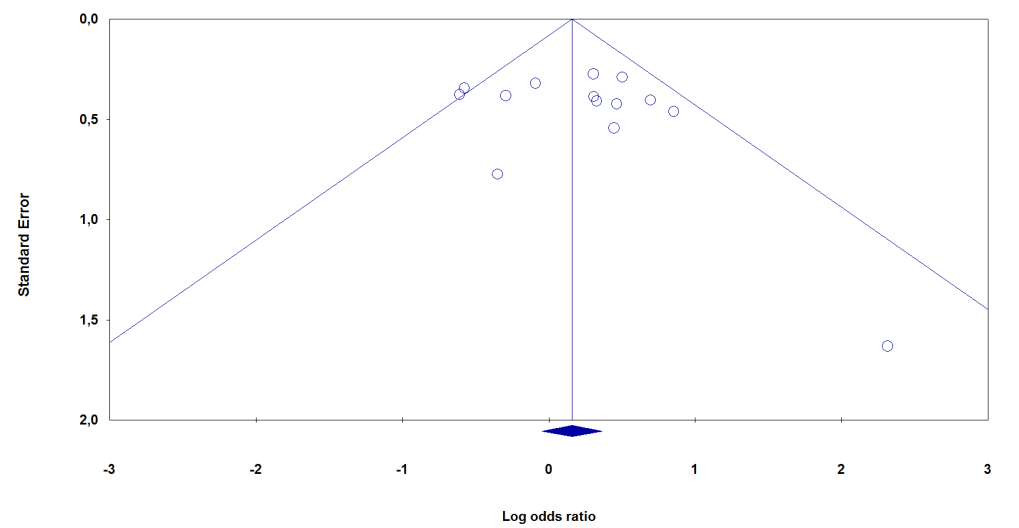  B |
| --- | --- |
| Begg’s test (p-value)= 0.485  Egger’s test (p-value)= 0.372 | Begg’s test (p-value)= 0.856  Egger’s test (p-value)= 0.226 |
| **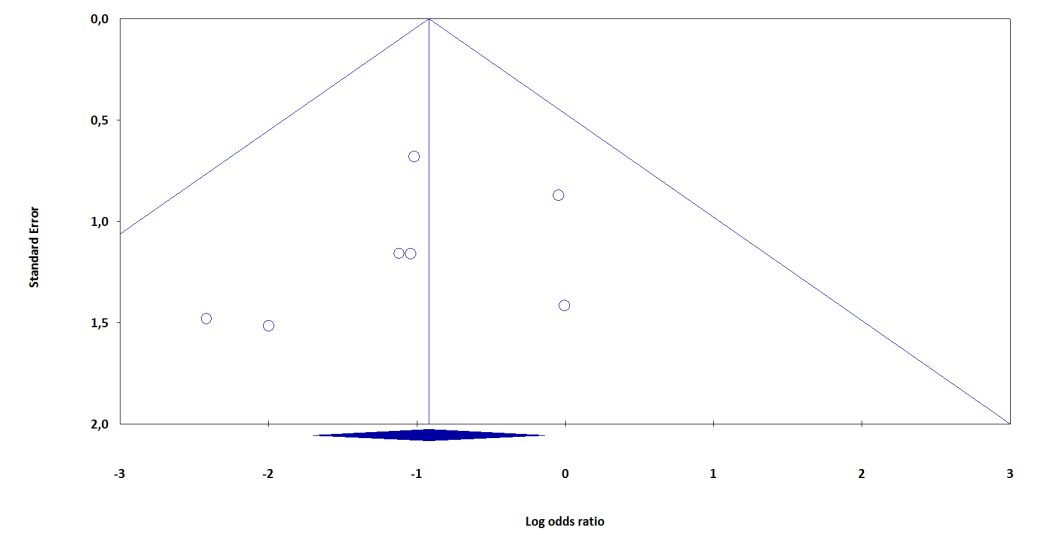**  C | 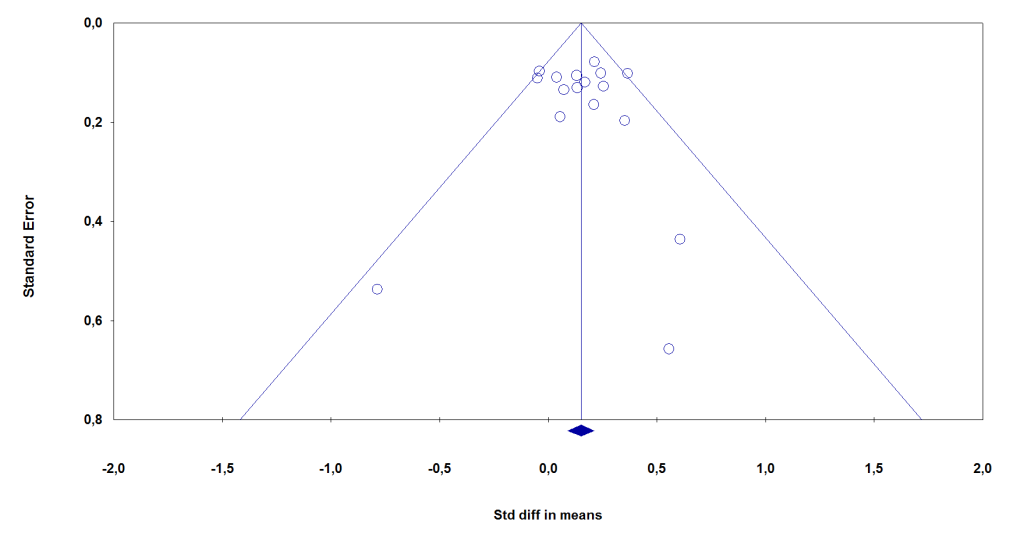  D |
| Begg’s test (p-value)= 0.147  Egger’s test (p-value)= 0.203 | Begg’s test (p-value)= 0.371  Egger’s test (p-value)= 0.491 |
| 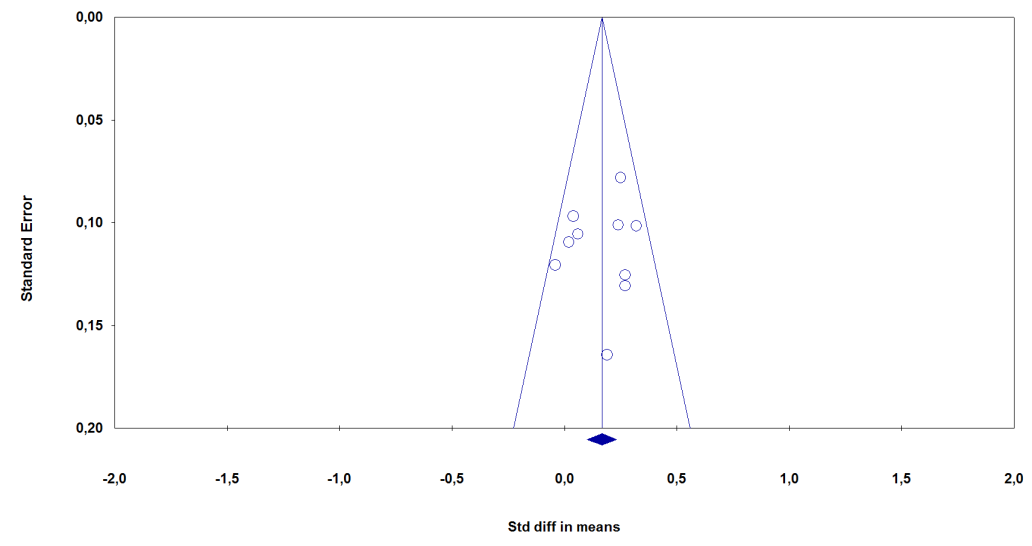  E | 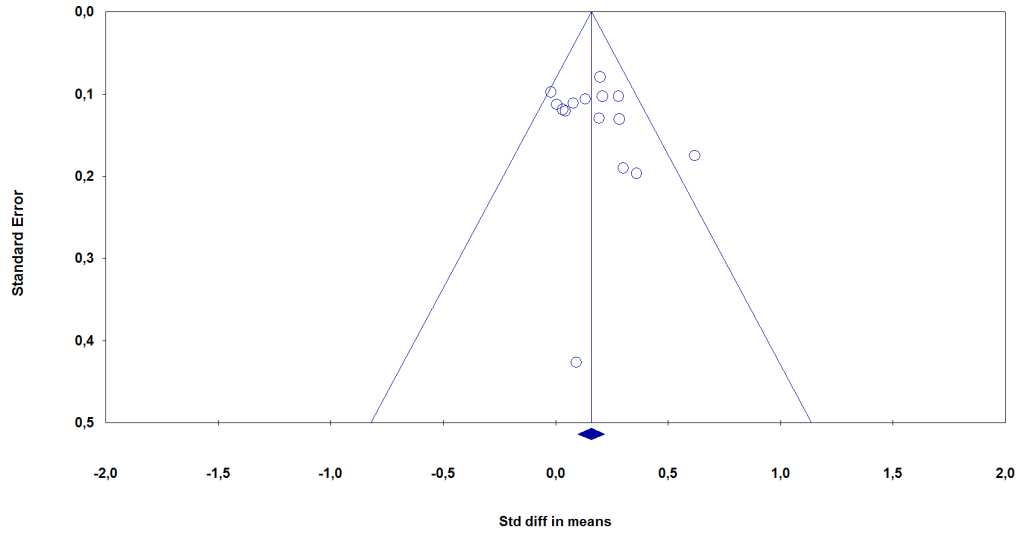  F |
| Begg’s test (p-value)= 0.266  Egger’s test (p-value)= 0.357 | Begg’s test (p-value)= 0.128  Egger’s test (p-value)= 0.127 |
| G | H |
| 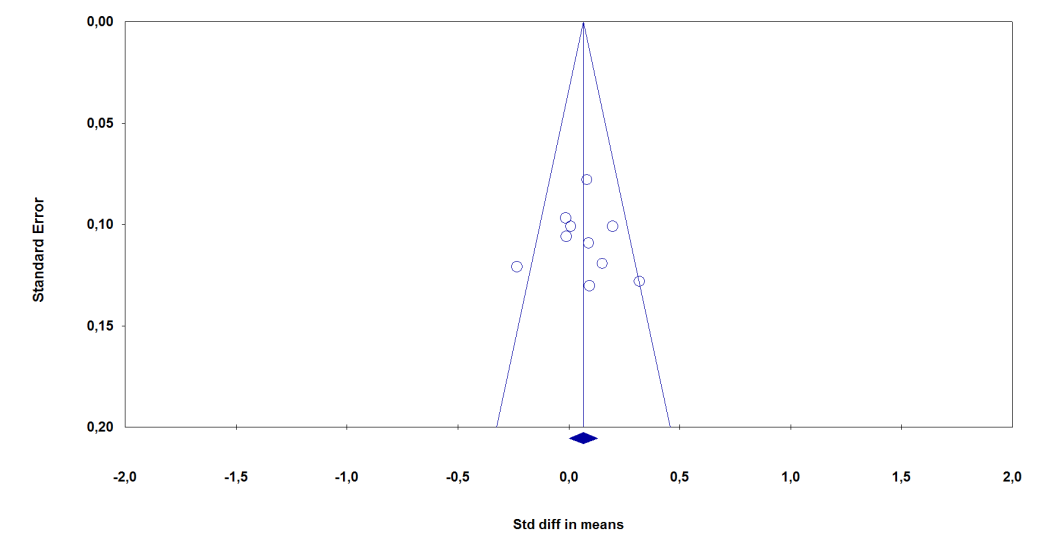Begg’s test (p-value)= 0.429  Egger’s test (p-value)= 0.424 | 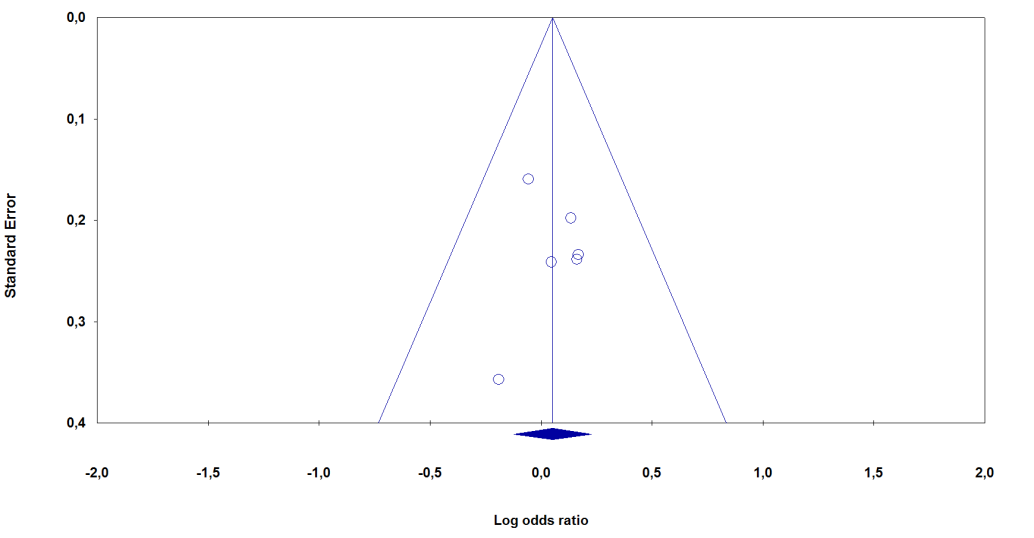  Begg’s test (p-value)= 0.425  Egger’s test (p-value)= 0.485 |
| 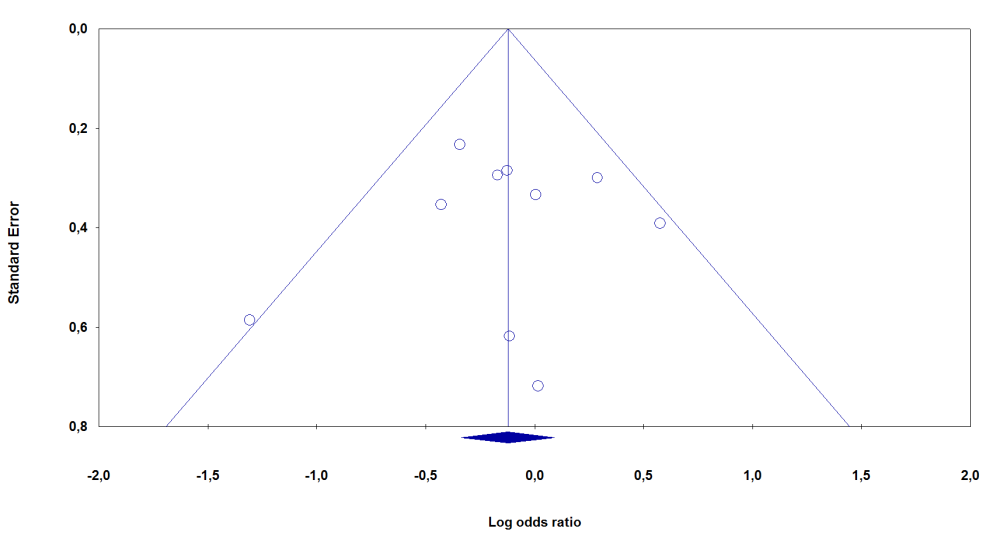  I | 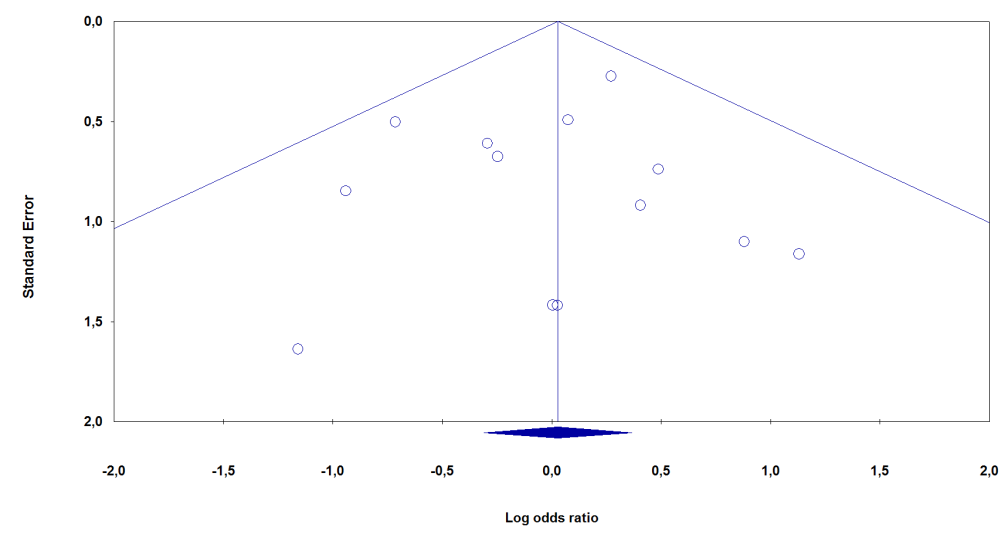  J |
| Begg’s test (p-value)= 0.266  Egger’s test (p-value)= 0.410 | Begg’s test (p-value)= 0.500  Egger’s test (p-value)= 0.348 |

**Figure S10** Funnel plots, Egger’s test and Begg and of study outcomes A. All-cause discontinuation outcome; B. Discontinuation due to adverse events; C. Discontinuation due to lack of efficacy; D. Efficacy on cognitive function; E. Efficacy on global change; F. Efficacy on neuropsychiatric symptoms; G. Efficacy on functional ability; H. Proportion patients with any adverse event; I. Proportion patients with severe adverse events; J. Mortality.
